# Combined inhibition of EZH2 and CDK4/6 perturbs endoplasmic reticulum-mitochondrial homeostasis and increases antitumor activity against glioblastoma

**DOI:** 10.1038/s41698-024-00653-3

**Published:** 2024-07-25

**Authors:** Thomas Freitag, Philipp Kaps, Justus Ramtke, Sarah Bertels, Emily Zunke, Björn Schneider, Anne-Sophie Becker, Dirk Koczan, Daniel Dubinski, Thomas M. Freiman, Felix Wittig, Burkhard Hinz, Mike-Andrew Westhoff, Hannah Strobel, Franziska Meiners, Daniel Wolter, Nadja Engel, Sascha Troschke-Meurer, Wendy Bergmann-Ewert, Susanne Staehlke, Annabell Wolff, Florian Gessler, Christian Junghanss, Claudia Maletzki

**Affiliations:** 1 Department of Medicine, Clinic III –Hematology, Oncology, Palliative Medicine, Rostock University Medical Center, University of Rostock, Rostock, Germany; 2 Institute of Pathology, Rostock University Medical Center, University of Rostock, Rostock, Germany; 3 Department of Immunology, Rostock University Medical Center, University of Rostock, Rostock, Germany; 4 Department of Neurosurgery, Rostock University Medical Center, University of Rostock, Rostock, Germany; 5 Institute of Pharmacology and Toxicology, Rostock University Medical Center, University of Rostock, Rostock, Germany; 6 Department of Pediatrics and Adolescent Medicine, University Medical Center Ulm, Ulm, Germany; 7 Institute for Biostatistics and Informatics in Medicine and Aging Research (IBIMA), Rostock University Medical Center, University of Rostock, Rostock, Germany; 8 Department of Oral and Maxillofacial Surgery, Facial Plastic Surgery, Rostock University Medical Center, University of Rostock, Rostock, Germany; 9 Oscar Langendorff Institute of Physiology, Rostock University Medical Center, University of Rostock, Rostock, Germany; 10 Department of Pediatric Oncology and Hematology, University Medicine Greifswald, Greifswald, Germany; 11 Core Facility for Cell Sorting & Cell Analysis, Laboratory for Clinical Immunology, Rostock University Medical Center, University of Rostock, Rostock, Germany; 12 Institute for Cell Biology, University Medical Center Rostock, Rostock, Germany

**Keywords:** Cancer metabolism, Cell death

## Abstract

Here, we show that combined use of the EZH2 inhibitor GSK126 and the CDK4/6 inhibitor abemaciclib synergistically enhances antitumoral effects in preclinical GBM models. Dual blockade led to HIF1α upregulation and CalR translocation, accompanied by massive impairment of mitochondrial function. Basal oxygen consumption rate, ATP synthesis, and maximal mitochondrial respiration decreased, confirming disrupted endoplasmic reticulum-mitochondrial homeostasis. This was paralleled by mitochondrial depolarization and upregulation of the UPR sensors PERK, ATF6α, and IRE1α. Notably, dual EZH2/CDK4/6 blockade also reduced 3D-spheroid invasion, partially inhibited tumor growth *in ovo*, and led to impaired viability of patient-derived organoids. Mechanistically, this was due to transcriptional changes in genes involved in mitotic aberrations/spindle assembly (*Rb, PLK1, RRM2, PRC1, CENPF, TPX2*), histone modification (*HIST1H1B, HIST1H3G*), DNA damage/replication stress events (*TOP2A, ATF4*), immuno-oncology (*DEPDC1*), EMT-counterregulation (*PCDH1*) and a shift in the stemness profile towards a more differentiated state. We propose a dual EZH2/CDK4/6 blockade for further investigation.

## Introduction

Central Nervous System WHO grade 4 glioblastoma (GBM), is a highly malignant brain tumor with a poor prognosis^1–3^. This is attributable to intratumoral heterogeneity, epigenetic modifications, cell cycle dysregulation, and stemness, among other factors. Although heterogeneity and stemness are undruggable targets, epigenetic modifications and cell cycle dysregulation have gained increasing attention.

The epigenetic modifier Enhancer of zeste homolog 2 (EZH2) is part of polycomb repressor-complex 2 (PRC2). PRC2 is responsible for inducing epigenetic changes in the DNA *via* trimethylation of lysine 27 at histone 3 (H3K27me3), resulting in gene silencing and heterochromatin formation. This process contributes to the regulation of genes that are critical for the control of cell cycle checkpoints, differentiation, and adhesion. EZH2 plays an essential role in stemness, invasion, and temozolomide resistance in GBM^4,5^. The proposed mechanism includes binding to Heterochromatin Protein 1 Binding Protein 3 (HP1BP3) and epigenetic co-activation of Wnt Family Member 7B WNT7B^6^. Moreover, EZH2-mediated suppression of PTEN leads to AKT/mTOR signaling activation and increased proliferation and migration of GBM cells^7^. Cumulatively, these findings highlight the role of EZH2 as a relevant oncogene whose expression correlates with poor patient outcomes^5,8^. The observation of relatively homogenous expression of EZH2 in GBM^9^ renders this oncogene a potential therapeutic target. Indeed, several preclinical studies have confirmed successful targeting of GBM cells by specific EZH2 inhibition^10–13^. These agents either inhibit the EZH2 methyltransferase activity or disrupt the protein-protein interactions among the PRC2 subunits^14^. GSK126, just like the FDA-approved Tazemetostat, belongs to the S-adenosylmethionine competitive small-molecule inhibitors of the methyltransferase activity. GSK126, in particular, is highly selective against EZH2 compared to other methyltransferases and effectively reduces GBM cell self-renewal and invasiveness^5,15–17^. Dysregulation of the cell cycle is another hallmark of GBM that leads to hyperproliferation. In fact, the CDK4/6-Retinoblastoma axis is dysregulated in ~80% of all GBM cases^18^. *CDKN2A* is of particular interest owing to its crucial role in cell cycle regulation. It encodes the CDK4/6 inhibitor p16^INK4a^ which guides the transition into the S-phase, and p14^ARF^ protein, which enhances p53 activity. In GBM, *CDKN2A* is often deleted or dysfunctional^19,20^. Accordingly, the pharmacological targeting of CDKs is a focus of ongoing research. In our previous studies, we confirmed the therapeutic activity of the CDK inhibitors, dinaciclib and abemaciclib^21,22^. Abemaciclib is a CDK4/6 inhibitor approved by the FDA and EMA for the treatment of advanced HR-positive breast cancer^23,24^. It binds to the ATP cleft of the target CDKs with greater selectivity for CDK4 than for CDK6. Abemaciclib also has activity against other kinases, including glycogen synthase kinase 3α/β and calmodulin-dependent protein kinase II α/β/γ^25^. Antitumoral effects against glioblastoma include mitochondrial dysfunction, impaired invasiveness, and induction of the DNA damage response^21,22^.

Given the relevance of epigenetic modifications and cell cycle dysregulation in GBM, we applied an integrative treatment approach involving multiple preclinical models here. We focused on EZH2 and CDK4/6 as druggable targets. Our results demonstrated a massive impairment of viability and invasion of 2D- and 3D-GBM cultures upon dual EZH2 and CDK4/6 blockade with the two blood-brain barrier penetrating agents GSK126 and abemaciclib^10,26^. Mechanistically, we identified altered gene expression, reversal of stem-like characteristics, induction of cell stress *via* impaired mitochondrial function, and disruption of the endoplasmic reticulum (ER)-mitochondrial homeostasis. These effects cumulatively enhance cell death in patient-derived GBM models in vitro and *in ovo*.

## Results

### EZH2 is overexpressed in GBM and combined EZH2/CDK4/6 inhibition synergistically boosts cell death in GBM cells

**Fig. 1:**
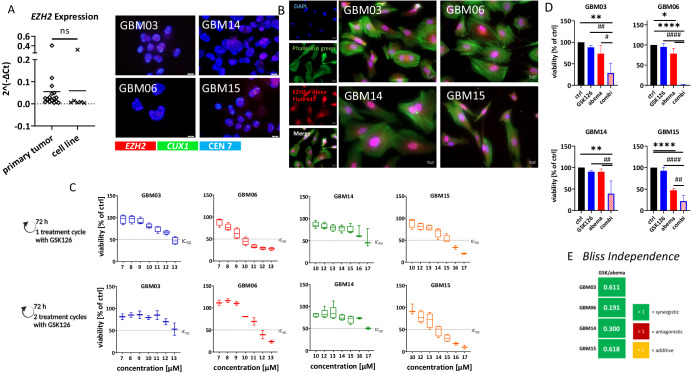
EZH2 as a target in glioblastoma. **A** EZH2 expression levels (qPCR) in primary tumors and patient-derived GBM cell lines (left) (ns not significant), as well as FISH analysis (right) to detect *EZH2* in GBM cells. FISH was performed using the ZytoLight FISH Cytology implementation Kit according to the manufacturer’s protocol using the following probes: ZytoLight SPEC CUX1 (green)/EZH2 (red)/CEN 7 (blue) Triple Color Probe. The criteria for gene amplification were defined as the presence of either four (or more) gene signals or more than 2.5 times as many gene signals as centromere signals of the related chromosome. **B** protein abundance in four patient-derived GBM cell lines. Representative immunofluorescence images are shown. Nuclei were stained with DAPI (blue), actin filaments with Phalloidin green (green), EZH2 using an Alexa Fluor® 647-conjugated secondary antibody (red). Single-channel and merged fluorescence are presented (scale bar: 20 μm). **C**–**E** Influence of GSK126 alone and in combination with abemaciclib on the viability of GBM cells. **C** Concentration-response relationships to GSK126 for one (72 h) and two (2 × 72 h) treatment cycles to determine the IC_20_ and IC_50_ concentrations. **D** Presented is the response to GSK126 (10 µM), abemaciclib (1 µM) and the combination (**C**, **D**) of both after 2 treatment cycles with 72 h each; n = 3, mean ± s.d. One-way ANOVA (Tukey’s multiple comparisons test); *p < 0.05; **p < 0.01; ****p < 0.0001 (comparison between control and test group); ^#^p < 0.05; ^##^p < 0.001; ^####^p < 0.0001 (comparison between testing groups). **E** The Bliss independence model was used to assess additive or synergistic effects in the combination compared to each monotherapy after 2 × 72 h of treatment. Created with Biorender.com.

The gene expression profile of *EZH2* was first studied in primary tumors and patient-derived cell lines (Fig. 1A). *EZH2* transcripts were detected in all primary tumors, and their expression was preserved after in vitro culture (Fig. 1A, left). FISH analysis of four freshly established GBM cell lines demonstrated amplification of *EZH2* in GBM03, 06, and 15 (Fig. 1A, right). The only exception was GBM14, in which *EZH2* was not amplified (Fig. 1A, right). High EZH2 expression was confirmed at the protein level. No cell-line-specific differences were observed (Fig. 1B), hence, in GBM14, we found a slight discrepancy between gene and protein expression. The cells were further subjected to basic characterization by molecular pathology for potential correlation analysis with the mutational profile of classic tumor suppressor genes (*i.e. TP53*, *PIK3CA*, and *PTEN*, Table 1). Sensitivity to the EZH2 inhibitor GSK126 was investigated (Fig. 1C).

**Table 1:** Clinical patients data, including basic molecular characterization of GBM samples

Patient ID	Gender/age	Diagnosis	*MGMT* status	*IDH1/2* status	Mutation			
					*TP53*	*KDR*	*PIK3CA*	*PTEN*
GBM03	m/81	GBM CNS WHO grade 4	Unmethylated	wt	R273H (VAF: 48.6 %) R248Q (VAF: 50.3 %) P72R (VAF: 99.6 %)	Q472H (benign, VAF: 35.0 %)	wt	wt
GBM06	m/71	GBM CNS WHO grade 4	Methylated	wt	G244A (VAF: 99.9 %) P72R (VAF: 100.0 %)	wt	I391M (VAF: 30.6 %)	wt
GBM14	m/63	GBM CNS WHO grade 4	Unmethylated	wt	V173L (VAF: 58.6 %) P72R (VAF: 58.3 %)	Q472H (benign, VAF: 54.0 %)	wt	D252Y (VAF: 100.0 %)
GBM15	m/40	GBM CNS WHO grade 4	Unmethylated	wt	P72R (VAF: 99.9 %)	wt	wt	wt
GBM26	w/72	GBM CNS WHO grade 4	Methylated	wt	N/A	N/A	N/A	N/A
GBM31	w/61	GBM CNS WHO grade 4	Unmethylated	wt	N/A	N/A	N/A	N/A
GBM37	w/74	GBM CNS WHO grade 4	Unmethylated	wt	N/A	N/A	N/A	N/A

*CNS* central nervous system, *VAF* variant allele frequency.

GBM cells were incubated with GSK126 for either a single 72-h period or for two consecutive 72-h cycles. Although slightly varying responses to GSK126 were observed between individual cell lines (Fig. 1C), there were no significant differences between single and double applications. Overall, GBM14 cells were less sensitive to GSK126 than the other cell lines. This cell line had no *EZH2* amplification (Fig. 1A, right) and harbored a missense mutation in *PTEN* (D252Y, Table 1), which was not detected in other cases.

Next, we focused on combination strategies using the CDK4/6 inhibitor abemaciclib as a combination partner (Fig. 1D, E). Cyto-FISH and sequencing analysis identified *CDK4* amplification in three of four cases (GBM03/06/15) and a partial *CDKN2A* deletion in GBM06 (Supplementary Fig. 1), thus providing a for using this compound as a combination approach.

Both compounds were used at clinically relevant^15,27,28^ concentrations, referring to ~IC_20_ values (10 µM GSK126, 1 µM abemaciclib, except: GBM15, because of a higher sensitivity to abemaciclib), and administered twice (2 × 72 h). This co-administration enhanced the antitumor activity of each monotherapy (Fig. 1D). Notably, in GBM06, less than 2% of viable cells were detected after the combination treatment (p < 0.0001 *vs*. control). In GBM03, GBM14, and GBM15 the effects were less pronounced; however, synergism was still achieved, as determined by the Bliss Independence model (Fig. 1D, E).

### Dual EZH2/CDK4/6 blockade affects target protein abundance and induces cell-line-specific changes in morphology

**Fig. 2:**
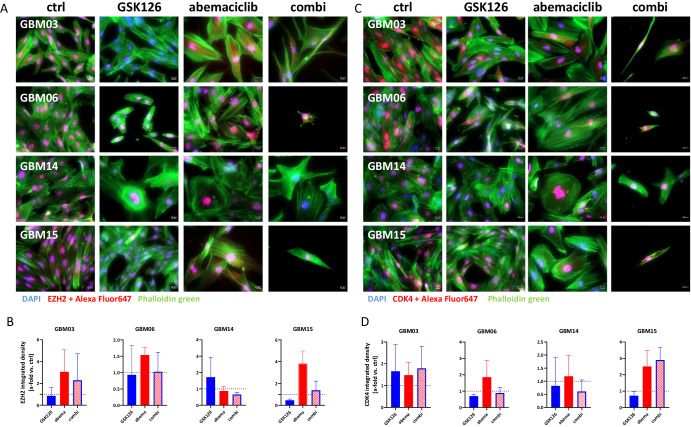
Treatment-induced changes in the EZH2 and CDK4 protein expression. Immunofluorescence for assessment of **A**, **B** EZH2 and **C**, **D** CDK4 protein levels in 2D cell cultures with or without treatment (GSK126 10 µM, abemaciclib 1 µM, combination, 2 × 72 h). Nuclei were stained with DAPI (blue), actin filaments with Phalloidin green (green), EZH2/CDK4 using an Alexa Fluor® 647-conjugated secondary antibody (red). **B**, **D** The quantification is presented as the x-fold change (integrated density) relative to the control (DMSO), which was set to =1 (dotted line); n = 3, mean ± s.d. Kruskal–Wallis test (Dunn’s multiple comparisons test).

We then examined the effect of EZH2 and CDK4/6 blockade on target protein abundance and the cytoskeleton in residual cells (Fig. 2).

GSK126 resulted in a slight reduction in EZH2 protein levels in GBM03, GBM06, and GBM15 cells. An inverse trend was observed in GBM14, in which EZH2 protein levels were higher after treatment (Fig. 2A, B). Interestingly, abemaciclib alone had the opposite effect, with higher EZH2 levels in three of the four cell lines. In combination treatment, no changes were observed, except for GBM03. In this cell line, abemaciclib alone induced the highest level of EZH2 which was only partially reversed by the combination.

The abemaciclib target protein CDK4 was slightly higher in GBM03, regardless of treatment (Fig. 2C, D), most likely because of *CDK4* gene amplification (see Supplementary Fig. 1). Similarly, abemaciclib led to the accumulation of CDK4 in residual GBM06 and GBM15 cells. This compensatory effect was abolished by the combination in two out of four cases (GBM06, GBM14).

Assessment of cell morphology revealed dramatic changes upon treatment, which were particularly evident in the combination (Fig. 2A, C). Residual cells showed a flattened (GBM03, GBM14) and occasionally spindle-shaped structures (GBM03 and GBM15), with perinuclear stress fibers (GBM15), suggesting increased cell stress (Fig. 2A, C). Cell shrinkage, accompanied by lamellipodia/filopodia formation, was also observed in GBM06 and GBM15.

### Combined EZH2/CDK4/6 inhibition boosts cell stress and reverses stemness characteristics in GBM cells

**Fig. 3:**
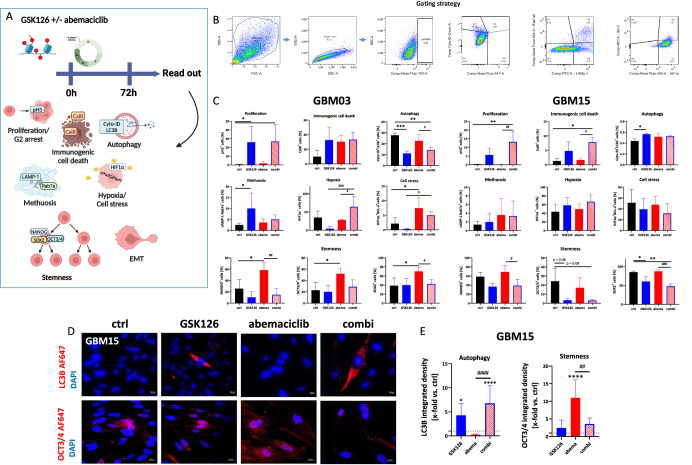
Influence on autophagy, proliferation, cell stress, methuosis, and stemness. **A** Schematic overview of the treatment strategy and its impact on different cell death and escape mechanisms. Created with Biorender.com. **B** Gating strategy for flow cytometry quantification. **C** Quantitative analysis of the investigated markers using spectral flow cytometry. Given is the % of positive cells after exposure to GSK126 (10 µM), abemaciclib (1 µM) and the combination of both (treatment: 1 × 72 h); n = 3–5, mean ± s.d. One-way ANOVA (Tukey’s multiple comparisons test); *p < 0.05; **p < 0.01; ***p < 0.001; (comparison between control and test group) ^#^p < 0.05; ^##^p < 0.001; ^###^p < 0.001 (comparison between testing groups). **D**, **E** Immunofluorescence for assessment of autophagy (LC3B) and stemness (OCT3/4). Expression levels of LC3B and OCT3/4 were determined after 2× 72 h with or without treatment (GSK126 10 µM, abemaciclib 1 µM, combination) **D** Representative images of GBM15 cells. Nuclei were stained with DAPI (blue), autophagocytotic cells with an Alexa Fluor® 647 anti-LC3B antibody (red) or an Alexa Fluor® 647 anti-OCT3/4 antibody (red). **E** The quantification is presented as the x-fold change (integrated density) relative to the control (DMSO), which was set to =1 (dotted line); n = 3, mean ± s.d. One-way ANOVA (Tukey’s multiple comparisons test); *p < 0.05; ****p < 0.0001 (comparison between control and test group) ^#^p < 0.05; ^##^p < 0.001; ^####^p < 0.0001 (comparison between testing groups).

To further analyze the underlying mechanisms of the response in more detail, GBM03, GBM06, and GBM15 cells were included in a multi-parameter flow cytometric analysis (Fig. 3 and Supplementary Figs. 2 and 3). GBM14 was excluded due to the lower sensitivity to treatment. The analysis was done after 1 × 72 h treatment. The gating strategy is illustrated in Fig. 3B. In some cases, immunofluorescence was done after 2 × 72 h to study effects after repeated treatment. The results are described below.

In all cell lines, the number of pH3^+^ cells was higher after GSK126 mono- and combination treatment (GBM03: p < 0.05 *vs*. control; GBM15: p < 0.01 *vs*. control), indicating mitosis and cell cycle arrest in the G2/M phase. Similarly, calreticulin (CalR) translocation was more abundantly detected in the combination (GBM15: p < 0.05 *vs*. the control and abemaciclib). However, effects on autophagy were cell-line-specific. In GBM03 cells, all treatments reduced the number of autophagocytotic cells (defined as Cyto-ID^+^LC3b^+^), whereas in GBM15 cells, only GSK126 enhanced autophagocytosis compared to controls. After 2 × 72 h treatment, the effects on autophagocytosis increased in this cell line (Fig. 3D, E). As can be seen in Fig. 3D (upper part), LC3B levels were significantly elevated after EZH2 inhibition and remained high in the combination (p < 0.0001 vs. ctrl, Fig. 3E). Thus, double treatment enhanced autophagocytosis, which was primarily driven by GSK126. Autophagic flux was then examined with and without the addition of bafilomycin A1 followed by SQSTM1 staining (Supplementary Fig. 4). SQSTM1 levels increased after GSK126 mono- and combination treatment (p < 0.05 and p < 0.001 vs. ctrl, respectively), confirming EZH2 inhibitor-driven autophagocytosis. Autophagic flux inhibition by bafilomycin A1 slightly decreased SQSTM1 levels. However, SQSTM1 levels remained high in cells treated with GSK126, indicating complex stress responses after EZH2 blockade^29^. Indeed, apoptosis- and autophagy-independent cell death methuosis (defined as LAMP1^+^Rab7a^+^) was strongly induced. In GBM03 and GBM15 cells, the numbers of LAMP1^+^Rab7a^+^ cells increased upon GSK126 exposure (GBM03: p < 0.05 vs. ctrl). For GBM06, the treatments had no effect on methuosis. Still, the cell stress-associated protein HIF-1α was higher after treatment in all cell lines, and even significantly increased in GBM03 by the combination (p < 0.0001 *vs*. GSK126, p < 0.05 *vs*. abemaciclib). Co-expression with BCL-2 was higher exclusively in GBM03 after abemaciclib monotherapy and combination treatment (abemaciclib: p < 0.05 *vs*. ctrl; combination: p < 0.05 *vs*. GSK126), whereas it tended to decrease in GBM06 and GBM15 (Fig. 3C and Supplementary Fig. 2A).

In addition, the impact of both treatment strategies on cellular stemness was investigated. We focused on NANOG, OCT3/4, and SOX2, which are associated with treatment resistance, cell regulation, and inhibition of differentiation^30–32^. In GBM03, abemaciclib led to a significant increase in NANOG^+^ and SOX2^+^ cells, which was counter-regulated by the combination (p < 0.01 and p < 0.05 *vs*. abemaciclib, respectively), mainly due to GSK126. A similar trend was observed for OCT3/4, although it did not reach statistical significance. No clear changes were seen in GBM06 cells (Supplementary Fig. 2A). However, GSK126 combined with abemaciclib tended to reduce stemness markers compared with the controls. Similar effects were observed in GBM15, where the combination resulted in reduced numbers of stem-like cells (NANOG: p < 0.05, SOX2: p < 0.001 *vs*. abemaciclib, respectively). In all cases, this combination effect was driven by GSK126. To further confirm this, immunofluorescence was performed with GBM15 cells after 2 × 72 h and thus prolonged treatment (Fig. 3D, E, and Supplementary Fig. 2B, C). OCT3/4 significantly increased after abemaciclib (p < 0.0001 *vs*. ctrl). The combination reduced OCT3/4 expression to levels equivalent to GSK126 monotherapy (p < 0.01 vs. abemaciclib, Fig. 3D, E). The presence of A2B5, a highly specific marker of stemness and glioma initiation that is widely expressed among individual GBM subtypes^33,34^, was then examined. Of note, in GBM15, A5B5 expression was significantly upregulated by abemaciclib alone (p < 0.05 vs. ctrl), but downregulated by the combination (Supplementary Fig. 2B, C). A comparable pattern was seen in GBM06 (Supplementary Fig. 2C) that confirms the reversal of stem-like features in residual GBM cells after combined EZH2/CDK4/6 blockade. Besides, a classical colony formation assay with GBM06 and GBM15 showed impaired colony-forming ability of residual cells in the combination (Supplementary Fig. 2D). Additional quantification of the EMT markers E-cadherin, N-cadherin, vimentin, and SNAI/Slug revealed no significant effect of either treatment on any of these markers, suggesting that EMT is of minor relevance, at least after 1× 72 h short-term treatment. In support of this, the EMT-related E-cadherin “switch” was only observed in GBM03, but not in the other cell lines (Supplementary Fig. 3). Considering that the E-cadherin switch may not be essential in GBM^35^, and to test whether prolonged treatment triggers the transition, GBM15 and GBM06 cells were stained for the mesenchymal marker vimentin (Supplementary Fig. 3B, C). With this analysis, we indeed identified increased vimentin levels only after abemaciclib monotherapy (GBM06: p < 0.001 vs. ctrl; GBM15 p < 0.0001 vs. ctrl). GSK126 had no effect on this mesenchymal marker and successfully counteracted the abemaciclib-induced upregulation in the combination.

To sum up these findings, the addition of GSK126 to abemaciclib (I) enhances the beneficial cytotoxic effects of CDK4/6 blockade and (II) neutralizes its undesirable “side” effects, i.e. stemness and EMT.

### Combined EZH2/CDK4/6 inhibition disrupts endoplasmatic reticulum-mitochondrial homeostasis

**Fig. 4:**
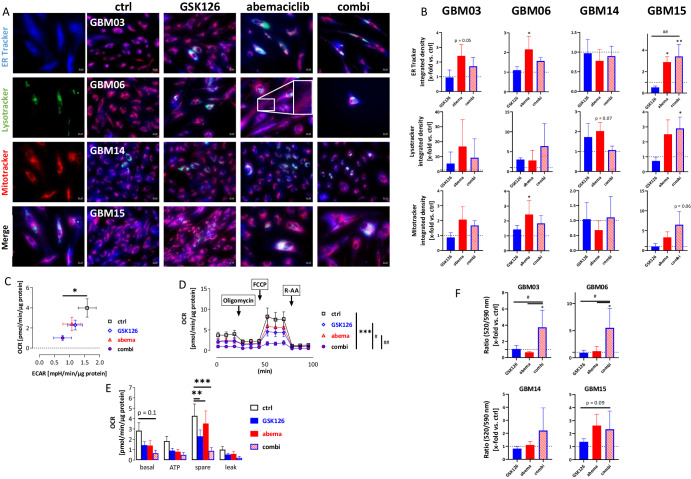
Impact on ER stress, lysosomes, mitochondria, and extracellular flux analysis. **A**, **B** ER stress (ER-tracker), acidic compartments (LysoTracker), and mitochondrial function (Mitotracker) were examined in 2D-cultured GBM cells with or without treatment (GSK126 10 µM, abemaciclib 1 µM, combination, 2× 72 h) by immunofluorescence staining as described in the “Methods” section (ER- [blue], Lyso- [green], and Mitotracker [red]). **A** Single-channel and merged fluorescence are presented (representative images are shown, scale bar A: 20 μm, n = 3). **B** Quantification determined as the x-fold change (integrated density) in relation to the control, which was set to be =1 (dotted line); Kruskal–Wallis test (Dunn’s multiple comparisons test); *p < 0.05; **p < 0.01 (comparison between control and test group); ^##^p < 0.01 (comparison between testing groups); Complex cellular stress responses were seen in all cell lines, with individual effects of the treatments. **C**–**E** The oxygen consumption rate (OCR) and extracellular acidification rate (ECAR) of GBM15 cells were measured using a Seahorse XFe24 Analyzer. **C** OCR and ECAR decreased after treatment with GSK126 (10 µM), abemaciclib (1 µM), or a combination of both. One-way ANOVA (Tukey’s multiple comparisons test); *p < 0.05 (comparison between control and test group). **D** Mitochondrial stress test was applied with injection of 1.5 µM oligomycin, 1.5 µM FCCP, and 0.5 µM antimycin A and rotenone, each. Kruskal–Wallis test (Dunn’s multiple comparisons test); ***p < 0.001 (comparison between control and test group); ^#^p < 0.05; ^##^p < 0.01 (comparison between testing groups). **E** To determine the basal respiration (basal), ATP-dependent respiration (ATP), Spare respiratory capacity (spare), and proton leak (leak) were determined. One-way ANOVA (Tukey’s multiple comparisons test); **p < 0.01;***p < 0.001 (comparison between control and test group). Reduction of basal OCR and spare respiratory capacity was shown, indicating massively reduced cellular fitness. **F** JC-10 Mitochondrion Membrane Potential Assay. One-way ANOVA (Tukey’s multiple comparisons test); *p < 0.05 (comparison between control and test group); ^#^p < 0.05 (comparison between testing groups); **B**–**F** n = 4; mean ± s.d. All experiments were done after 2 × 72 h treatment with GSK126 (10 µM), abemaciclib (1 µM), or the combination of both.

The above data indicated increased cellular stress following the dual blockade of EZH2 and CDK4/6 with GSK126 and abemaciclib. Since EZH2 is essential for the regulation of spindle assembly and CDKs are involved in mitochondrial function, we investigated whether these important cellular mechanisms were also affected by both treatments (Fig. 4).

First, the unfolded protein response (UPR), which is activated under conditions of endoplasmic reticulum (ER) stress, was examined (Fig. 4A, B). This was observed in three out of four cell lines after treatment with abemaciclib or the combination, as indicated by an increase in ER-Tracker fluorescence intensity in the ER compared to controls (Fig. 4A). Concurrently, the number of lysosomes increased after treatment, notably in all treatment groups (*i.e*. monotherapy and combination compared to controls) which was likely because of lysosomal accumulation in cellular vesicles. In GBM15, the effects were more pronounced, reaching significance in the combination group (p < 0.05 *vs*. ctrl).

After treatment, a peculiar mitochondrial “chain-like” network was observed in individual residual cells, indicating impaired mitochondrial function. This was primarily driven by abemaciclib and partly offset by the combination (please see Fig. 4A, GBM06: white box). The only exception was GBM15, where the number of mitotracker-positive cells increased, suggesting increased membrane potential compared to the controls (Fig. 4A, B).

To obtain more detailed information on the effects of the treatments on mitochondrial respiration and glycolysis, a Seahorse Extracellular Flux (XF24) analyzer was used with GBM15 cells (Fig. 4C–E). Following treatment with abemaciclib, GSK126, or their combination, both OCR and ECAR exhibited a significant reduction compared to the controls (p < 0.05 *vs*. ctrl; Fig. 4C). This decline reveals a metabolic shift towards a less energetically favorable state.

**Fig. 5:**
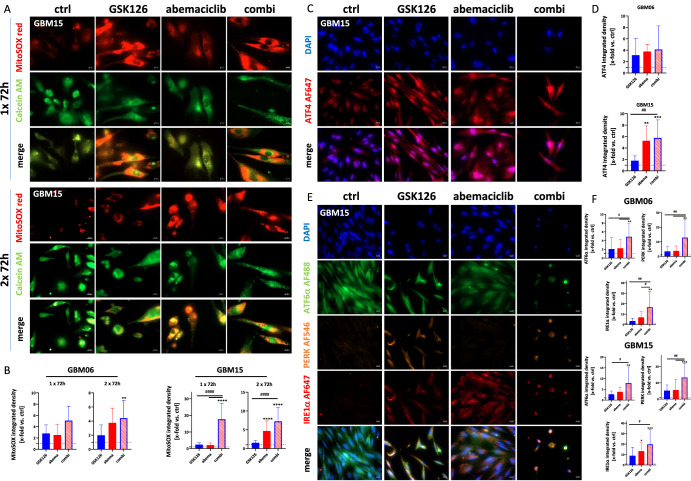
Immunofluorescence for analysis of the unfolded protein response and mitochondrial impairment. **A**, **B** MitoSOX™ red was used as an indicator of ROS production in GBM cells. Cells were counterstained with Calcein AM [green] for the spatial distribution. **A** Single-channel and merged fluorescence are presented (representative images for GBM15 are shown, scale bar A: 20 μm). Analysis was done after 1× and 2 × 72 h treatment with GSK126 (10 µM), abemaciclib (1 µM), or a combination of both. **B** Quantification determined as the x-fold change (integrated density) in relation to the control, which was set to be =1 (dotted line); n = 3, mean ± s.d. Kruskal–Wallis test (Dunn’s multiple comparisons test); **p < 0.01; ****p < 0.0001 (comparison between control and test group); ^####^p < 0.0001 (comparison between testing groups); There was a significant time-dependent increase in ROS production when GBM cells were exposed to the GSK126/abemaciclib combination. **C**, **D** ATF4 immunofluorescence for detection of the integrated stress response in GBM cells (GBM06, GBM15). **C** Single-channel and merged fluorescence are presented (representative images for GBM15 are shown, scale bar A: 20 μm). Analysis was done after 2× 72 h treatment with GSK126 (10 µM), abemaciclib (1 µM), or a combination of both. Nuclei were stained with DAPI (blue), ATF4 was detected by using an Alexa Fluor® 647 anti-ATF4 antibody. **D** The quantification is presented as the x-fold change (integrated density) relative to the control (DMSO), which was set to =1 (dotted line); n = 3, mean ± s.d. One-way ANOVA (Tukey’s multiple comparisons test); **p < 0.01; ***p < 0.001 (comparison between control and test group); ^##^p < 0.01 (comparison between testing groups). **E**, **F** Immunofluorescence for detection of the unfolded protein response in GBM cells (GBM06, GBM15). **E** Single-channel and merged fluorescence is presented (representative images for GBM15 are shown, scale bar A: 20 μm). Analysis was done after 2× 72 h treatment with GSK126 (10 µM), abemaciclib (1 µM), or a combination of both. Nuclei were stained with DAPI (blue), antibody staining included the following: Alexa Fluor® 488 anti-ATF6α, Alexa Fluor® 546 anti-PERK, Alexa Fluor® 647 anti-IRE1α. **F** The quantification is presented as the x-fold change (integrated density) relative to the control (DMSO), which was set to =1 (dotted line); n = 4, mean ± s.d. One-way ANOVA (Tukey’s multiple comparisons test); **p < 0.01; ***p < 0.001 (comparison between control and test group); ^#^p < 0.05; ^##^p < 0.01; (comparison between testing groups). Combined treatment with GSK126 and abemaciclib enhanced cellular PERK and IRE1α levels and confirmed the induction of a UPR in GBM cells.

Employing a Mito Stress test enabled the characterization of basal respiration and ATP production-dependent respiration (Fig. 4D). Spare respiratory capacity, defined as the difference between maximal and basal respiration, suggestive of the cellular ability to respond to stress and adjust its energy demand, was significantly inhibited by both compounds (p < 0.0001 *vs*. ctrl; p < 0.05 *vs*. GSK126, and p < 0.01 *vs*. abemaciclib Fig. 4D). Notably, the combined application of these compounds exhibited an even more pronounced impact on the spare respiratory capacity parameter, underscoring a synergistic effect in modulating the cellular stress response and energy adaptation (Fig. 4E). This was also confirmed by the JC-10 fluorescent mitochondrial membrane potential assay (Fig. 4F). Mitochondrial membrane potential was significantly increased in GBM cells 03, 06, and 14 after dual EZH2/CDK4/6 blockade, confirming impaired mitochondria (Fig. 4F). To track the spatial and temporal occurrence of mitochondrial impairment, MitoSOX™ staining was performed on GBM06 and GBM15 cells after 1× and 2× 72 h incubation (Fig. 5A, B). Elevated levels of mitochondrial ROS were already detectable after a single treatment with GSK126 and abemaciclib (GBM15: p < 0.0001 vs. ctrl and GSK126 and abemaciclib). After two treatment cycles, mitochondrial ROS production was further enhanced (GBM06: p < 0.01 vs. ctrl; GBM15: p < 0.0001 vs. ctrl and GSK126). Likewise, levels of ATF4, the main effector of the integrated stress response (ISR), significantly increased in GBM15 cells (Fig. 5C, D). To confirm the above findings of a UPR under treatment and an associated ER stress, detailed analysis was then done for the three ER-resident sensors, inositol-requiring protein 1α (IRE1α), activating transcription factor 6α (ATF6α), and PRKR-like endoplasmic reticulum kinase (PERK, Fig. 5E, F). PERK and IRE1α were barely detectable in untreated controls, but protein levels increased significantly after mono- and combination treatment. ATF6α levels also increased after treatment and were again highest in the combination. Hence, the UPR was confirmed in GBM06 and GBM15 cells. Cumulatively, these data point to disrupted ER-mitochondrial homeostasis as the leading cause of GBM cell death following dual EZH2 and CDK4/6 blockade with GSK126 and abemaciclib.

### Combined EZH2/CDK4/6 inhibition reduces the invasion capability in GBM spheroids

**Fig. 6:**
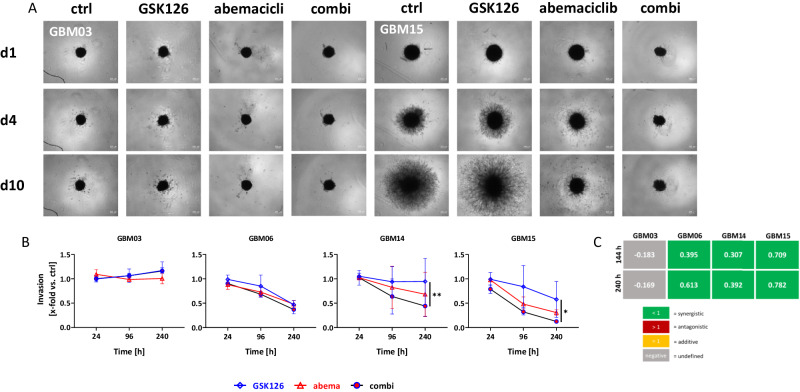
Invasion capability of the different cell lines and calculation of synergistic effects. **A** Representative images of the invasion under treatment with GSK126 (10 µM), abemaciclib (1 µM), the combination of both, or with vehicle control after the spheroids were embedded in matrigel. Images were taken on day 1, 4, and 10. The spheroids were treated on day 0 and day 3 (2 × 72 h in total). **B** Increase in the spheroid area compared to the spheroid area of control spheroids on the respective day, which was set to 1. n = 3; mean ± s.d; Two-way ANOVA (Tukey’s multiple comparisons test); *p < 0.05; **p < 0.01 (comparison between testing groups). **C** The Bliss independence model was used to assess whether the combination showed additive or synergistic effects compared to each individual monotherapy. Created with Biorender.com.

To analyze whether the observed effects could be transferred to a more clinically relevant model, we generated 3D spheroids and monitored their ability to invade the Matrigel over a period of 10 days. The spheroid size was measured relative to the size of the controls on each day (Fig. 6 and Supplementary Fig. 5).

After 10 days, nearly all GBM control spheroids exhibited strong invasive behavior, albeit to varying degrees. The only exception was GBM03, in which spheroids, neither control nor treated, barely invaded the surrounding matrix. In the other cell lines, invasive ability was reduced by all treatments, with the most pronounced inhibition observed in the combination treatment. Accordingly, synergistic anti-invasive effects were confirmed mathematically (Fig. 6C).

Thus, the dual blockade of EZH2 and CDK4/6 effectively inhibited cellular invasion in a clinically relevant spheroid model.

### Combined EZH2/CDK4/6 inhibition alters gene expression in GBM15 spheroids

**Fig. 7:**
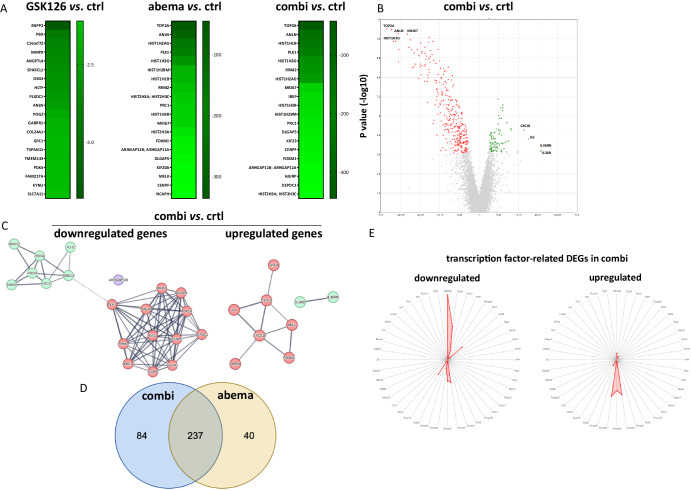
Treatment of GBM cells with abemaciclib in combination therapy induces changes in genes involved in mitotic aberrations/spindle assembly, histone modification, DNA damage/replication stress events, and immuno-oncology. **A** Microarray analysis with resulting heatmap displaying the top differentially expressed genes across all conditions, sorted by fold change. **B** Volcano plot representing all up- and downregulated genes between controls and the combination group (GSK126 10 µM, abemaciclib 1 µM, 2× 72 h). **C** Protein-protein interaction network of gene clusters top 20 downregulated and upregulated genes between control cells and the combination. Isolated nodes were removed from the network. The edges indicate both functional and physical protein interactions and the line thickness of network edges indicates the strength of data support, where minimum interaction score was set to high confidence (0.700) and FDR < 0.01. The clustering was performed using the Markov Cluster Algorithm (MCL) with an inflation parameter set to 3. **D** The Venn diagram visualizes the shared and exclusively DEGs between the combination treatment group and the abemaciclib treatment group. **E** Radar chart illustrates the exclusively DEGs associated with transcription factors under combination treatment.

Treatment-related changes at the molecular level were determined by gene expression and functional enrichment analyses. GBM15 spheroids were used for analyses. The gene expression datasets were filtered with an adjusted false discovery rate p-value of <0.01 and an FC ± >2 to obtain differentially expressed genes (DEGs, Fig. 7A).

GSK126 treatment had no significant effect on gene expression, whereas abemaciclib treatment resulted in 318 DEGs, of which 34 were upregulated and 284 were downregulated. Key downregulated DEGs included *TOP2A, ANLN, PLK1, RRM2, PRC1*, and histone-related genes (*H1-5, H3C15, H2BC14, H3C8*, and *H3C2*), with fold changes ranging from −101 to −336. Functional enrichment analysis revealed two clusters of downregulated genes (Fig. 7), highlighting pathways such as mitotic prometaphase *(NCAPH, NCAPG, PLK1, CENPF)*, Polo-like kinase mediated events *(PLK1, CENPF)*, and mitosis *(PLK1, TOP2A)*.

The combination treatment had the most substantial impact, identifying 419 DEGs (93 upregulated and 326 downregulated) (Fig. 7B). Hence, the synergistic effect of dual EZH2/CDK4/6 blockade was confirmed on a transcriptional level. Downregulated genes included essential mediators of cell proliferation, including *TOP2A, ANLN, PLK1, RRM2, MKI67*, and histone-related genes, with fold changes between −344 and −86 (Fig. 7A). Two clusters (Fig. 7C) were identified in the protein-protein interaction network of downregulated genes, sharing similarities with the abemaciclib-treated cell cluster. The enriched pathways included PLK-mediated events and the mitotic cell cycle. The second cluster comprised histone proteins enriched in different pathways.

Overall, 237 DEGs were shared between single abemaciclib- and combination-treated spheroids (Fig. 7D, Jaccard index: 0.66, = moderate to high similarity between these two gene sets). Exclusive alterations in the combination included genes such as *GPC4, SLC14A1*, and genes involved in matrix stiffening, immunosuppression, and invasion (e.g., *COL12A1*, *MMP9, CSF1*, and *PDGFRB)*. The massive downregulation of invasion-associated genes confirms our above finding of a suppressed EMT phenotype after dual EZH2/CDK4/6 inhibition. Knowing that multiple genes are involved in this complex process, we further analyzed EMT-related gene signatures (Supplementary Fig. 3D). Among the EMT-related genes, *matrix metallopeptidase 9, ITGA6*, and its associated downstream target *FOXM1*, were the most significantly downregulated genes. *Protocadherin 1 (PCDH1*), which binds to SMAD3 and acts as a tumor suppressor, was significantly upregulated (p < 0.0001 vs. ctrl). ITGA6 and FOXM1 are of particular interest because of their dual roles in GBM, i.e. induction of EMT and support of stemness. Therefore, we analyzed the effect on stemness in more detail using the StemChecker online tool^36^. A direct comparison between abemaciclib monotherapy and combination therapy revealed a shift in the stemness profile (Fig. 7E). We identified 84 exclusively downregulated DEGs, with 29 of which were associated with cancer stem cells. A more detailed analysis of the gene expression profile identified a switch in transcription factor-associated DEGs from NANOG to Smad molecules. The downregulated genes were primarily associated with the NANOG transcription factor, whereas the upregulated genes were associated with the Smad family members 2, 3, and 4, indicative of a loss of pluripotency in favor of a more differentiated state and correspondingly less invasive behavior. Finally, essential genes of the Wnt/β-catenin pathway, i.e. *CTNNB1, TCF*, and *PLAU* were significantly downregulated by the combination (Supplementary Fig. 2E).

### Combined EZH2/CDK4/6 inhibition slightly impairs tumor growth in the CAM model

**Fig. 8:**
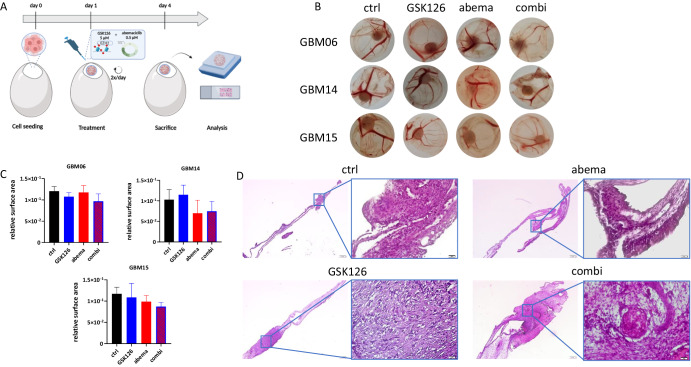
Impact of treatments on *in ovo* cultured tumors. **A** Schematic representation of the experimental set-up. Created with Biorender.com. **B** Macroscopic images of tumors from GBM cell lines under different treatment conditions (GSK126 5 µM, abemaciclib 0.5 µM, twice a day, for a total of 3 days). **C** Quantitative evaluation of the relative surface area of tumor size. n = 4–5; mean ± s.d. **D** HE images of GBM15 *in ovo* cultures from controls or treatment with GSK126, abema or the combination. Representative images at low and high magnification (4× and 40×, respectively), Scale bar = 200 µm, 5–6 eggs per condition.

Then, the semi in vivo CAM model was applied to GBM06, GBM14, and GBM15 (Fig. 8). GBM03 was excluded due to its reduced 3D-invasiveness and poor *in ovo* xenograft engraftment. In two of the three cases (GBM14, GBM15), the combination therapy slightly reduced the relative tumor size within the treatment period (Fig. 8B, C). However, in GBM06, tumor size was comparable between the control and treatment groups. Despite this, an alleged reduction in tumor vascularization was observed under treatment compared to that in the control, suggesting a potential impact on tumor angiogenesis and vasculature (Fig. 8B, D). All the chicks were alive after the experiment. This could be considered an indirect indication that the inhibitors were not lethal at the concentrations and over the time period tested. In summary, we have confirmed the therapeutic potential of dual EZH2 and CDK4/6 blockade in clinically relevant GBM models.

### Improved treatment response in preclinical PDO models

**Fig. 9:**
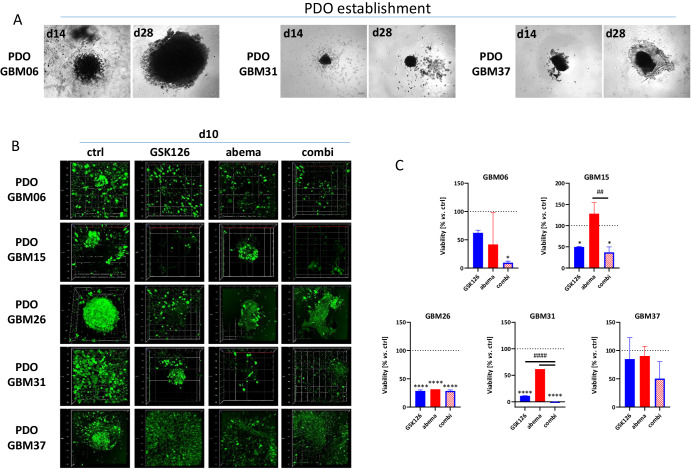
Generation of patient-derived organoids for targeted treatment. **A** PDOs were generated from frozen tissue of n = 5 individual GBM cases. Representative phase contract images of PDOs at day 14 and day 35 of culture in defined PDO medium. **B** Calcein AM staining was done to visualize the 3D-structure of PDOs and to assess the viability *via* confocal laser scanning microscopy (Z-stack analysis). Therefore, growing PDOs (at ~day 35 of culture) were cut into defined pieces (~200 µm), transferred into 96-well ULA plates, and exposed to treatments. Confocal laser scanning microscopy was done after 10 days of treatment (including 2× 72 h, +4 days follow-up). **C** Quantitative analysis of PDO viability was assessed after 10 days of treatment (including 2 × 72 h, +4 days follow-up) using the CellTiter 3D Glo assay as described in the “Methods” section. n = 2; mean ± s.d. One-way ANOVA (Tukey’s multiple comparisons test); *p < 0.05; ****p < 0.0001 (comparison between control and test group); ^##^p < 0.01; ^###^p < 0.001 (comparison between testing groups).

Patient-derived organoids (PDOs) were next established from frozen tumor material of five individual patients and exposed to GSK126, abemaciclib, or a combination of both (Fig. 9A). As we only had material from GBM06 and GBM15, but not from GBM03 and GBM14, tumor material from three additional cases with histologically proven GBM was included (clinical information is provided in Table 1).

The response to GSK126, abemaciclib or its combination was individual between the PDOs, but the overall response rates could be confirmed in these clinically relevant primary tumor models (Fig. 9B, C). In GBM06, we obtained essentially the same synergistic effect after dual EZH2/CDK4/6 blockade as obtained before in the cell line (p < 0.05 vs. ctrl). In GBM15, antitumoral effects in the combination were mainly attributable to GSK126, while abemaciclib alone had no cytotoxic effect. Instead, PDOs tended to be more viable. However, this negative effect was antagonized in the combination and viability significantly decreased (p < 0.05 vs. ctrl). In the additional three cases not previously tested but included in this preclinical mini-ex vivo study, a mixed response was seen. GBM26 was sensitive to either EZH2 or CDK4/6 blockade, but, no enhanced effect was seen with the combination. In GBM31, viability decreased to <1% in the combination (p < 0.0001 vs. ctrl and abemaciclib). PDOs from GBM37 were less sensitive, but dual EZH2/CDK4/6 blockade reduced viability to approximately 50%.

Taken together, these data confirm the successful application of two different targeted agents, i.e. GSK126 and abemaciclib, in preclinical GBM models and provide further evidence for the potential of dual EZH2/CDK4/6 blockade to control this disease.

### Application of the FDA-approved EZH2i tazemetostat and effects in combination with abemaciclib

Finally, we decided to transfer our concept of dual EZH2/CDK4/6 blockade to the FDA-approved EZH2 inhibitor tazemetostat, which is currently used in several clinical trials in different clinical settings. Single-agent application at different doses identified a dose-response pattern similar to GSK126, with, however, higher IC_50_ values (>10 µM; Supplementary Fig. 6A). The addition of abemaciclib as a combination partner to GBM06 and GBM15 was synergistic, but the antitumor effects were lower compared to the GSK126/abemaciclib combination (Supplementary Fig. 6B). We applied tazemetostat at 10 µM which corresponds to the doses of GSK126. Although this dose was insufficient to yield substantial GBM cell death, cell stress may be induced and was studied here. As ROS production and ER stress were identified as the main reasons for GSK126/abemaciclib-mediated cell death, we also focused on these mechanisms. In line with our previous findings, mitochondrial ROS production increased after two rounds of treatment, and reached significance in the combination (GBM06: p < 0.05; GBM15: p < 0.01 vs. control; Supplementary Fig. 6C, D). ER stress, as indicated by higher ER-tracker fluorescence intensity, also increased compared to controls and monotherapies. The same effects were seen on mitochondria (Supplementary Fig. 6E, F). This suggests that the response mechanisms are similar for both compounds (GSK126, tazemetostat). Still, the weaker cytotoxic activity in combination with tazemetostat compared to GSK126 suggests a delayed effect of the FDA-approved EZH2 inhibitor.

## Discussion

In this study, we targeted epigenetic alterations and the dysregulated cell cycle in glioblastoma. Therefore, we applied an integrative treatment approach to a panel of 2D, 3D, *in ovo*, and PDO models to identify the mechanisms underlying dual EZH2 and CDK4/6 inhibition. EZH2 is overexpressed in GBM and associated with treatment resistance, stemness, and poor outcomes^37^. Several preclinical studies have already confirmed the antitumor activity of EZH2 inhibitors, including tazemetostat and GSK126, which inhibit the methyltransferase activity^5,10,13,16,38,39^. In addition, S-phase perturbations and G2/M transitions controlled by CDKs are common in GBM; however, single targeting with specific inhibitors is usually ineffective^40^, making combinations of different agents inevitable. Abemaciclib demonstrated efficacy in our previous studies, where we reported the formation of small vacuoles and myelin figures, the induction of methuosis-like processes, and mitochondrial dysfunction in GBM models^21,22^.

In the first part of this study, we confirmed the expression and amplification of *EZH2* in patient tumor samples and patient-derived cell lines. In one case (GBM14), the gene expression status was slightly different from the protein level. No gene amplification was detected, but protein levels were as high as in the other GBM cell lines. We speculate that decreased internalization and turnover of the EZH2 protein may explain this discrepancy, which was already shown for other cancer-specific oncogenes^41^. Molecular analysis revealed alterations in the cell cycle control genes *CDK4* and *CDKN2*A. In 2D cell cultures, we demonstrated that GSK126 and abemaciclib alone impaired the viability of our newly established GBM cultures. Notably, the combination of the two agents synergistically enhanced the effects of monotherapies and significantly impaired cell viability. EZH2 was largely unaffected by GSK126 in three out of four cases, and an even higher protein level was detected after treatment in GBM14, which supports the hypothesis of a decreased protein internalization, and eventually other, so far unknown, intrinsic mechanisms that stabilize EZH2 in these cells. Also, the relatively low sensitivity to GSK126 is not unexpected. Another study of *Her2*-amplified breast cancer showed less benefit of Her2-directed therapy when the tumors showed this heterogeneity or mismatch between gene amplification and protein overexpression^41^. In addition, the minor impact of GSK126 on EZH2 protein abundance seems puzzling at first; however, knowing that GSK126 inhibits the enzyme activity of EZH2 without affecting its expression at the transcriptional level explains this finding. EZH2 levels are dependent on different signaling pathways, including Wnt/β-Catenin, which plays a crucial role in regulating cell proliferation and differentiation^6,42^. Activation of the Wnt/β-Catenin pathway can increase EZH2 expression, contributing to the regulation of genes involved in the cell cycle and stem cell properties.

*CDK4* amplification and/or *CDKN2A* loss are reliable biomarkers of CDK inhibitors^43–45^. Here, *CDK4* amplification, accompanied by partial *CDKN2A* loss was detected in three of four cases, resulting in high basal protein abundance, which was not reduced after CDK4/6 blockade. Still, the combination of GSK126 and abemaciclib slightly reduced the CDK4 protein levels in the remaining cells.

The enhanced cytotoxic effects of the combination were cell-line-specific. The cell-line GBM06 responded best, as only a few residual cells were observed after dual EZH2/CDK4/6 blockade, notably in 2D-, 3D-spheroid, and clinically relevant PDO models. Molecularly, GBM06 was the only tumor with *MGMT* promoter methylation and a *PIK3CA* I391M mutation, which does not alter kinase activity, but eventually increases cell proliferation and viability^46^. Whether *MGMT* promoter methylation serves as a biomarker for dual EZH2/CDK4/6 blockade is currently unknown. Since EZH2 overexpression is frequently associated with a lack of response to adjuvant chemo-radiotherapy^47^ and a predictor of poor outcome, this may provide a strategy to identify patients likely to benefit from this approach. To date, this conclusion is speculative and must be confirmed using a larger patient cohort. In addition, the lack of effect on key cellular characteristics after a single 72-h treatment requires prospective consideration, as the primary cause of cell death in the few remaining cells of GBM06 has been incompletely characterized. So far, we speculate that a very rare subpopulation within the bulk cells was less sensitive to treatment. Although the bulk tumor cell mass was effectively killed by dual EZH2/CDK4/6 blockade, our flow cytometric approach may have missed or unidentified potential effects on this rare subpopulation.

The PTEN status is another marker to consider, as H3K27me3, which is activated by EZH2, blocks *PTEN* transcriptional activation^48^. Conversely, targeting EZH2 impairs mTORC1 activity through an indirect mechanism that upregulates *PTEN* expression in breast cancer patients^49^. In our study, a missense mutation was detected in GBM14, which is the least sensitive cell line here.

The concept of a combined EZH2/CDK blockade is not completely new^50^. In a previous study, the HOTAIR - EZH2 inhibitor AQB and the CDK4/6 inhibitor palbociclib induced G1-arrest in GBM cells and inhibited Wnt/β-catenin signaling more effectively than single drugs^50^. In support of this, we identified accelerated cell stress, G2-arrest, decreased cell invasion, and a shift in the stemness profile upon dual EZH2/CDK4/6 blockade. The mitochondrial fitness was severely impaired. This was confirmed *via* (I) increased ROS production; (II) mitochondrial hyperpolarization; and (III) activation of the ISR and UPR signaling. Thus, the blockade of essential target molecules destroyed the mitochondrial reserve capacity as they were no longer able to respond to additional energy demands. This contributed to the ATP crisis, accelerated HIF-1α-mediated cell stress, CalR translocation, and ultimately cell death. Accordingly, ATF4, the main effector of the ISR, was significantly upregulated. Although ATF4 can act as both an activator and an inhibitor of transcription, we interpret this finding as a consequence of the massive activation of ISR for a prolonged period (i.e. 2× 72 h) that leads to cell death. In support of this, dual EZH2/CDK4/6 blockade-induced essential proteins of the UPR, including IRE1α, PERK, and ATF6α.

SQSTM1, which functions as a cargo protein involved in misfolded protein degradation *via* selective autophagy^29^, increased after EZH2 mono- and combination treatment. Expression levels were slightly affected by treatment with bafilomycin A1, a specific inhibitor of vacuolar H^+^-ATPase that prevents late-stage autophagy by inhibiting autophagosome/lysosome fusion^51^. Thus, autophagy is a consequence of sustained ER stress in our study. These data cumulatively identify disrupted ER-mitochondrial homeostasis as the leading cause of GBM cell death following GSK126 and abemaciclib combination treatment. Notably, these harmful effects on the mito-ER axis were seen across all cell lines – irrespective of the individual differences seen in terms of cell viability and cell death.

Another important finding of our study was the reversal of abemaciclib-induced upregulation of the stemness markers NANOG and OCT3/4 and the EMT markers N-cadherin and vimentin by the combination. This beneficial counterregulatory mechanism was primarily orchestrated by GSK126 and supports previous reports of an EZH2 inhibitor-mediated suppression of EMT and stemness through inhibition of H2K27-trimethylation of genes encoding EMT-regulating transcription factors^52^. Adding to this, we identified *ITGA6* and *FOXM1* as targets after the dual EZH2/CDK4/6 blockade. Both genes orchestrate the mesenchymal transition, promote GBM proliferation, invasion, and stemness, and were suppressed by the combination^53^. In contrast, the tumor suppressor *protocadherin 1*, which binds to SMAD3 and inhibits TGF-β1 signaling, was significantly upregulated, highlighting the therapeutic potential of this approach. In ¾ of the cell lines, we also observed an upregulation of EZH2 after abemaciclib monotherapy, which we interpret as an additional indicator of increased stemness in residual cells^4,5^. Therefore, the addition of an EZH2 inhibitor to abemaciclib (i) enhances the beneficial cytotoxic effects of CDK4/6 blockade and (ii) neutralizes its unwanted “side effects”, i.e. stemness and EMT. RNA profiling of GBM15 spheroids identified a switch in transcription factor-associated DEGs from NANOG to Smad molecules. Smad proteins are part of the TGFβ cascade, responsible for cell differentiation and development^54^. The altered expression profile indicates a loss of pluripotency in favor of a more differentiated state and correspondingly less invasive behavior. It also suggests changes in signaling pathways, i.e. cells in a Smad-dominated state may be less susceptible to certain growth signals or even more sensitive to therapies that directly or indirectly target these pathways. Hence, although GSK126 alone had no significant impact on the gene expression profile, its addition to abemaciclib dramatically altered the transcript levels of essential genes in a synergistic manner. Additionally to the described positive effects on EMT and stemness, an influence on DNA methylation and nucleosome accessibility (just like *HIST1H1B, HIST1H3G*) can be anticipated.

Hypoxia increases the expression of CDK2 and EZH2 by HIF-1α-dependent transcriptional activation. Finally, these global changes trigger a glycolytic shift, proteasome, and mitochondrial dysfunction^55,56^. Indeed, dual EZH2/CDK4/6 inhibition had global effects on genes primarily involved in mitotic aberrations/spindle formation (*Rb, PLK1, RRM2, PRC1, CENPF, TPX2*), DNA damage/replication stress events (*TOP2A, ATF4*), and immuno-oncology (*DEPDC1*). Notably, *RRM2* is associated with metabolic adaptation to temozolomide by facilitating dNTP availability, particularly in glioma stem-like cells, which contributes to chemoresistance and recurrence^57,58^. Inhibiting this molecule resulted in sensitization to radiation and synthetic lethality of GBM cells when combined with CHK inhibition^59^. Similar mechanisms are conceivable in our study as *RRM2* was dramatically downregulated after dual EZH2/CDK4/6 blockade. Besides, *TOP2A-*mediated transcriptional activity of β-catenin was abrogated in GBM cells and effectively prevented Wnt/β-catenin signaling activation and ultimately cell invasion^60^. In support of this, essential genes of the Wnt/β-catenin pathway, i.e. *CTNNB1, TCF*, and *PLAU* were significantly downregulated by the combination.

Additional effects after dual EZH2/CDK4/6 blockade include transcription of genes involved in migration, matrix remodeling, immunosuppression, and invasion (*e.g*. *COL12A1*, *MMP9, TSPAN11, CSF1, PLK4*, and *PDGFRB)*^61,62^. In particular, CSF1 and/or PLK4 inhibition can increase the number of tumor-infiltrating M1 macrophages in GBM^63,64^, thereby reversing immunosuppression. Specifically, CD163^+^ infiltration in *CDK4* amplified GBM^65^ may be reduced by this strategy, contributing to the rebalancing of immunogenicity. Also, genes involved in lipid metabolism (*SCD*), suppression of NF-κB signaling^66^ (*RCAN1*), and invasion inhibitors^67^ (*RND3*) were highly upregulated, revealing the mechanisms underlying the increased cell death upon dual EZH2 and CDK4/6 inhibition. Finally, we partially confirmed enhanced antitumor effects in short-term treatment *in ovo* and in the clinically relevant PDO models. In the latter, extensive disintegration of the dense and heterogeneous PDO structure was observed in 4/5 cases, and viability was dramatically reduced. The most pronounced effects were again observed in the combination.

In conclusion, our study supports the potential of dual EZH2 and CDK4/6 blockade as a promising therapeutic strategy for GBM, demonstrating superior efficacy compared to individual monotherapies across various models *via* perturbation of ER-mitochondrial homeostasis. These findings provide insights into the molecular mechanisms underlying the synergistic effects, suggesting potential biomarkers, such as alterations in the expression of *EZH2* and *CDK4/6*, *MGMT* promoter methylation status, activity levels of signaling pathways (*e.g*., Wnt/β-Catenin and PI3K/Akt), expression of mitotic genes (*e.g*., *Rb, PLK1, TPX2*), DNA repair genes (*e.g*., *EXO1* and *CKAP2L*), and changes in stemness markers (*e.g*., NANOG and OCT3/4) toward a more differentiated state, ultimately influencing GBM invasiveness. These markers could be used for patient stratification in future clinical applications. Finally, the synergistic effects observed following the combination of two FDA-approved agents, i.e. tazemetostat and abemaciclib, warrant further validation in larger cohorts of patients for subsequent translation into a clinical trial setting.

## Methods

### Patient samples, biobanking, establishment of 2D and 3D cultures

GBM tumor samples were obtained from patients during 5-Aminolevulinic acid (5-ALA) fluorescence-guided microsurgical resection at the Department of Neurosurgery of the Rostock University Medical Center, Germany. Written informed consent was obtained from all patients. All procedures involving patients were approved by the local Ethics Committee (Rostock University Medical Center, Ethics Registration ID: A2018-0167). The research related to human use has been complied with all relevant ethical regulations including the Declaration of Helsinki. The clinical patient information is listed in Table 1. The tumor specimens were processed under sterile conditions after resection. In brief, tumor pieces (3 × 3 × 3 mm) were viable cryopreserved (FCS, 10% DMSO) in liquid nitrogen. The remaining tumor fragments were snap-frozen for molecular analysis and were used for cell culture. Therefore, tumor pieces were homogenized using a cell strainer (100 µm) and seeded in 6-well plates containing DMEM/Hams F12 medium, supplemented with 20% FCS, 6 mM L-glutamine, and 1% penicillin/streptomycin (all from PAN-Biotech, Aidenbach, Germany) and incubated at 37 °C in a humidified atmosphere of 5% CO_2_. Continually growing cell cultures were serially passaged and regularly stored at low passages. For these experiments, we used four patient-derived cell lines: GBM03, GBM06, GBM14, and GBM15.

### *EZH2* expression

RNA isolation was performed using the RNeasy® kit (Qiagen, Hilden, Germany) following the manufacturer’s instructions and 1 µg of RNA was reverse transcribed into cDNA using a Random Hexamer Primer (50 ng in 20 µl). RT buffer complete, dNTP Mix, and reverase™ (Bioron, Römerberg, Germany) were added to finalize the transcription process. Afterward, 25 ng of cDNA was mixed with 0.65 µL of predesigned Taqman^TM^ gene expression assays 6-FAM-3ʹBHQ-1 *EZH2* (Applied Biosystems, Darmstadt, Germany), 0.65 µL of in-house 5-VIC-3ʹBHQ-1 *GAPDH* for normalization, 6.5 µL of master mix, and 3.2 µL of water. The reaction was performed in the light cycler Viia7 (Applied Biosystems), as described previously^68^. The expression level of each sample was determined by calculating the 2^−ΔCT^ (ΔCt = Ct_Target_ – Ct_Housekeeping gene_).

### Concentration-response curves

Cells (5000 cells/well) were seeded in 96-well plates (Greiner Bio-One, Kremsmünster, Austria) and incubated overnight. The test substances were dissolved in DMSO, and the stock solutions were diluted with DPBS. The final solvent concentration per compound used in the cell incubates was 0.1% (v/v) DMSO. Even when multiple test substances were added to the cells, the final concentration of DMSO in the incubates did not exceed 0.3% (v/v) DMSO. In all experiments, the incubation media of the vehicle- and substance-treated cells contained the same amount of solvent. Subsequently, 100 µL of medium containing increasing concentrations of GSK126 or abemaciclib was added and incubated for 72 h or two times 72 h. In some experiments, tazemetostat was used as a combination partner in doses equivalent to GSK126 (i.e. 10 µM). Cells were quantified after crystal violet staining using a Tecan reader Infinite M Plex (Tecan Group AG, Männerdorf, Switzerland, wavelength, 570 nm; reference: 620 nm).

### Immunofluorescence

3000 cells/well were seeded in µ-slides 18 wells (ibidi, Gräfelfing, Germany) and subjected to two consecutive 72-h treatments. Permeabilization and blocking were performed for 60 min using 0.5% Triton® X-100 and 2% BSA, respectively. Specific antibody (Ab) staining was done at RT using anti-human EZH2 (Cat# 14-9867-82) or anti-human CDK4 (Cat# MA5-41178, both from Thermo Fisher Scientific, stock: 1 mg/mL, 1:50, 2 h), followed by washing and incubation with the secondary antibody (Cat# 405322, anti-rabbit IgG (H + L), F(ab’)2 Fragment Alexa Fluor® 647, Biolegend, San Diego, CA, USA, 1:250, 60 min). After washing, phalloidin green staining was performed (Flash Phalloidin™ Green 488, Cat# 424201, BioLegend, 1:50, 20 min). Additionally, stemness and EMT markers were studied. Therefore, cells were incubated with monoclonal Abs: PE anti-human Nestin (Cat# 656805, Biolegend, 1:50, overnight), Alexa Fluor® 488 anti-human GFAP (Cat# 837508, Biolegend, 1:200, overnight), Alexa Fluor® 647 anti-human A2B5 (Cat# 150704, Biolegend, 1:200, overnight), Alexa Fluor® 594 anti-human Vimentin (Cat# 677804, Biolegend, 1:200, overnight), or Alexa Fluor® 647 anti-human OCT3/4 (Cat# 653710, Biolegend, 1:1000, overnight). To address the unfolded protein response, we used the following monoclonal Abs: Alexa Fluor® 647 anti-human ATF4 (Cat# sc-390063, Santa Cruz), Alexa Fluor® 488 anti-human ATF6α (Cat# sc-166659, Santa Cruz), Alexa Fluor® 546 anti-human PERK (Cat# sc-377400, Santa Cruz), and Alexa Fluor® 647 anti-human IRE1α (Cat# sc-390960, Santa Cruz), all 1:50, overnight. Nuclear staining was performed using DAPI (stock: 1.75 mg/mL; Thermo Fisher Scientific, 1:1750, 2 min), followed by microscopic examination (AxiovertA.1, Zeiss, Jena, Germany). In some experiments, bafilomycin A1 was used (5 nM in DMSO) as an autophagy inhibitor, followed by p62/sequestosome 1 (SQSTM1) staining using anti-p62 Alexa Fluor® 488 antibody (Cat# sc-28359, Santa Cruz, 1:50, overnight) as described above.

### Cellular stress response analysis using endoplasmic reticulum (ER) tracker, mitotracker, lysotracker & mitochondrial superoxide indicator

The experimental schedule was carried out in a manner similar to the previous immunofluorescence staining. Subsequently, MitoTracker Red CMXRos (Cell Signaling Technology, Danvers, Massachusetts, USA), LysoTracker ™ Green DND-26, and ER-Tracker™ Blue-White DPX (both from Thermo Fisher Scientific, Waltham, Massachusetts, USA) were stained according to the manufacturer’s instructions. Also, cells were stained with MitoSOX™ red (Thermo Fisher Scientific, 4 µM, Ex/Em 396 nm/610 nm) and counterstained with Calcein AM (Sigma Aldrich, 4 µM) to detect superoxide production as an indicator of cell stress. Images were obtained using a fluorescence microscope (AxiovertA.1, Zeiss, Oberkochen, Germany).

### Invasion & colony formation assay

10,000 cells/well were seeded into 96-well ultra-low attachment (ULA) plates (Greiner Bio-One, Kremsmünster, Austria) and all subsequent steps were done as previously described^21^. The colony formation assay was done as described^21^.

### Molecular analyses

Genomic alterations were analyzed using the Illumina Cancer Hotspot Panel (Illumina, Berlin, Germany) and Focus Panel, as described previously^69^. Gene-specific amplifications were studied by fluorescence in-situ hybridization (FISH) on fixed cells on a coated cytoslide (THARMAC cellspin, Wiesbaden, Germany) using the SHANDON cytospin3 centrifuge cell preparation system. FISH was performed using the ZytoLight FISH Cytology implementation Kit (ZytoVision, Bremerhaven, Germany) according to the manufacturer’s protocol using the following probes (all ZytoVision): ZytoLight SPEC CDKN2A/CEN 9 Dual Color Probe, ZytoLight SPEC CDK4/CEN 12 Dual Color Probe and ZytoLight SPEC CUX1/EZH2/CEN 7 Triple Color Probe. After hybridization and washing the sections were counterstained with DAPI/DuraText solution (ZytoVision) and analyzed using an Olympus BX53 fluorescence microscope system (Olympus, Hamburg, Germany) equipped with corresponding filter sets. The criteria for gene amplification were defined as the presence of either four (or more) gene signals or more than 2.5 times as many gene signals as centromere signals of the related chromosome.

### Microarray analysis

The total RNA of GBM15 spheroids was extracted, quantified, and expression profiling was performed using Applied Biosystem^TM^ Clariom^TM^ S arrays (formerly Affymetrix, Thermo Fisher Scientific) as described previously^22,70^. The gene expression datasets were filtered with an adjusted p-value < 0.01 and an FC ± >2 to identify differentially expressed genes (DEGs).

### Functional enrichment analysis

For enrichment and pathway analysis, differentially regulated genes were analyzed separately, as described previously^71^. To identify more specific biological pathways and processes^72^, the top 20 upregulated and downregulated genes were used to identify subgroups of correlated genes. Gene clusters were identified using STRING database version 12.0 (RRID:SCR_005223)^73^ and associated pathways and biological processes were assessed in each subgroup. Gene names were converted to Ensembl Gene IDs using the g:profiler g:Convert function^74^ prior to enrichment analysis.

### Spectral flow cytometry

Spectral flow cytometry was performed using three in-house multicolor panels as previously described^75,76^. Panel 1 was used to study proliferation and autophagy. Panel 2 examines viability, methuosis, and immune regulation, and Panel 3 provides information on immunogenic cell death, EMT, and stemness. Briefly, staining with antibodies, either targeting either extra- or intracellular antigens was done from 0.2 × 10^6^ cells. All procedures were performed using staining buffer (PBS, 2 mM EDTA, 2% BSA). The staining protocols for Panels 1 and 2 have been previously described^75^. Panel 3: Extracellular staining: Alexa Fluor405 mouse anti-human CalR (Cat# IC38981V-100UG, R&D Systems, Wiesbaden, Germany, 1:40), PE/Cyanine7 mouse anti-human CD325 (N-Cadherin, Cat# 350812, BioLegend, 1:25), APC-Vio770 mouse anti-human CD324 (E-Cadherin, Cat# 130-111-842, Miltenyi Biotec, Bergisch-Gladbach, Germany, 1:15), PerCP/Cyanine5.5 anti-human CD274 (PD-L1, Cat# 329738, BioLegend, 1:62.5). Cells were incubated for 20 min at room temperature, followed by permeabilization (BD Transcription Factor Buffer Set, BD Biosciences, New Jersey, USA, 1× Fix concentrate, 45 min, 4 °C) and antibody staining: VioB515 REA anti-human Vimentin (Cat# 130-129-207, Miltenyi Biotec, 1:31), PE mouse anti-human SNAI2/Slug (Cat# 564615, BD Biosciences, 1:50), BV421 anti-human SOX2 (Cat# 656114, BioLegend, 1:50), BV510 anti-human OCT3/4 (Cat# 563524, BD Biosciences, 1:50), and Alexa Fluor® 488 anti-human NANOG (Cat# 674206, BioLegend, 1:25). All subsequent steps were similar to those described previously^75^.

Measurements were done using a spectral flow cytometer (3L-Cytek^TM^ Aurora). The data were analyzed using SpectroFlo™ Version 3.2.1. and FlowJo™ Version 10.6.1, respectively. A detailed gating strategy for each analyzed marker is given in Supplementary Fig. 7.

### Cellular metabolic analysis

The oxygen consumption rate (OCR) and extracellular acidification rate (ECAR) were measured at the Seahorse Bioscience XFe24 Extracellular Flux Analyzer (Agilent Technologies, Inc., Waldbronn, Germany) according to the manufacturer’s instructions and as previously described^21^.

Briefly, cells were seeded in Seahorse 24XFe plates at a density of 10,000 cells/well and treated as described above. One hour before Seahorse XFe analysis, cells were washed and the medium was changed to XF base medium (pH 7.4) containing 10 mM glucose, 1 mM pyruvate, and 2 mM glutamine. The mitochondrial function was assessed by sequential injections of Seahorse XF Cell Mito Stress Test Kit compounds (Agilent Technologies, Inc.): via port A oligomycin (final concentration: 1.5 µM), port B FCCP (1.5 µM), and port C rotenone and antimycin A (0.5 µM), respectively. Basal respiration, ATP production-based respiration, proton leak, and spare capacity were calculated using the Seahorse XF Cell Mito Stress Test Report Generator (Agilent Technologies, Inc.). To normalize the data, the amount of protein/well was determined using the BCA Protein Assay Kit (Thermo Fisher Scientific). Lysates were generated with lysis buffer (50 mM HEPES, 150 mM NaCl, 1 mM EDTA, 1% [v/v] Triton® X-100, 10% [v/v] glycerol).

### JC-10 assay

The JC-10 Mitochondrion Membrane Potential Assay Kit (AAT Bioquest, Pleasanton, USA) was used. Therefore, 5000 cells per well were seeded in a 96-well microplate and treated as described previously, with each condition tested in triplicate. Afterward, cells were washed once with 100 µl warm PBS and 100 µl of DMEM containing 10 µM JC-10 was added. The cells were incubated for 45 min at 37 °C and 5% CO_2_. The supernatant was removed, and a wash step with 100 µl of PBS was performed. Next, fluorescence intensity (FI) was measured using a TECAN Plate Reader Infinite 200 Pro with excitation/emission wavelengths set at 490/525 and 540/590 nm. For data analysis, the fluorescence intensity of the blanks (cells without JC-10) was subtracted from the measured values. Then, the ratio Em 590/525 was calculated. Finally, the percentage increase in damaged mitochondria compared to the control was determined, and the mean values from the triplicates were calculated.

### Patient-derived organoids

All procedures involving patient-derived organoids (PDOs) were approved by the local Ethics Committee (Rostock University Medical Center, Ethics Registration ID: A2018-0167). Written informed consent was obtained from all patients. The experiments complied with all relevant ethical regulations including the Declaration of Helsinki. PDOs were established from n = 5 individual patients (Table 1) according to a protocol originally described by Jacob et al. with minor modifications^77^. Briefly, the tumor tissue was washed with ice-cold DPBS and transferred into Glioblastoma Dissection Medium (Hibernate A, 1% penicillin/streptomycin, 1% L-glutamine). Vital tumor tissue was separated from necrosis, hemorrhage, and normal brain tissue and dissected into small pieces (0.5–1 mm) using fine-spring dissection scissors. After lysing red blood cells and washing, tumor pieces were cultured in GBO medium (DMEM/F12, Neurobasal medium (1:1), 1× MEM-NEAAs solution, 1× N2 supplement, penicillin/streptomycin, L-glutamine, 1× NCS21 supplement, human insulin solution) on a 12-well suspension cell plate. The plate was incubated at 37 °C and 5% CO_2_ on an orbital shaker at 150 rpm. The media was replaced every three to four days. PDOs were used for subsequent experiments once they reached a rounded shape.

The viability of PDOs was assessed by staining with Calcein AM (4 µM) and analyzing them using a Zeiss Elyra 7 Confocal Laser Microscope. The viability was verified using the CellTiter 3D Glo Kit (Promega, Walldorf, Germany) in accordance with the manufacturer’s instructions. The luminescence signal was measured using the Tecan Plate Reader Infinite 200 Pro, with an integration time set to 1 s.

### Chorioallantoic membrane (CAM) assay

One million GBM06, GBM14, and GBM15 cells were transplanted onto the CAM of one-week-fertilized chicken eggs (LSL Rhein-Main, Dieburg, Germany). The cells were mixed 1:1 with serum-free medium and Matrigel (BD Biosciences, Franklin Lakes, NJ, USA). 24 h after transplantation, the treatment was started twice daily for 3 consecutive days. Substances were applied topically on the tumor area with 5 µL containing 5.0 µM GSK126, 0.5 µM abemaciclib, or the combination, with DMSO used as a control. After treatment, the tumor and surrounding CAM were extracted, embedded in paraffin, and cut into sections. The sections were stained with hematoxylin/eosin.

### Image processing

Image quantification was done by using the FIJI-ImageJ software as follows: Images were split into respective channels via ZEN software (Zeiss, Oberkochen, Germany). Staining intensity was then determined by integrated density profiles of the same size. For spheroid invasion, the total area was considered.

### Statistics

All values are presented as mean ± s.d. Statistical evaluation was performed using GraphPad PRISM software, version 8.0.2 (GraphPad Software, San Diego, CA, USA; RRID:SCR_002798). The criterion for significance was set at p < 0.05. All experiments were performed in biological triplicates, with two to three technical replicates. After proving the assumption of normality (Shapiro–Wilk test), one-way ANOVA (Dunnett’s or Holm-Sidak’s multiple comparisons test), two-way ANOVA (Tukey’s multiple comparisons test), or t-test was performed. If the normality test failed, the Kruskal–Wallis (Dunnett’s multiple comparisons test) or U-Test was performed. The following symbols were used: * *vs*. ctrl; # *vs*. monotherapy (GSK126, tazemetostat, or abemaciclib). The Bliss independence model was employed to evaluate the impact of combination effects. The analysis involved comparing the observed combination response with the predicted response, which was calculated based on the assumption of probabilistic independence. The Bliss independence interaction index was calculated to evaluate the synergistic or antagonistic effects of the drug combination.

### Supplementary information

Supplementary figures with legends beneath each one

## Data Availability

Gene expression data were loaded on the Gene Expression Omnibus (GEO) repository. The accession number is GSE268563.

## Code availability

Gene expression data were loaded on Gene Expression Omnibus (GEO) repository. The accession number is GSE268563 and the the code provides access to all data, without any restrictions (release date: Jun 01, 2024). The data were analyzed using Transcriptome Analysis Console TAC v4.0.3.

## References

[1] Banerjee, K. et al. Current approaches for glioma gene therapy and virotherapy. *Front. Mol. Neurosci.* **14**, 621831 (2021). 33790740 10.3389/fnmol.2021.621831 PMC8006286

[2] Silantyev et al. Current and future trends on diagnosis and prognosis of glioblastoma: from molecular biology to proteomics. *Cells* **8**, 863 (2019). 31405017 10.3390/cells8080863 PMC6721640

[3] Louis, D. N. et al. The 2021 WHO classification of tumors of the central nervous system: a summary. *Neuro. Oncol.* **23**, 1231–1251 (2021). 34185076 10.1093/neuonc/noab106 PMC8328013

[4] Liu, H. et al. EZH2 phosphorylation promotes self-renewal of glioma stem-like cells through NF-κB methylation. *Front. Oncol.* **9**, 641 (2019). 31380279 10.3389/fonc.2019.00641 PMC6652807

[5] Zhao, G. et al. Targeting EZH2 regulates the biological characteristics of glioma stem cells via the Notch1 pathway. *Exp. Brain Res.* **241**, 2409–2418 (2023). 37644332 10.1007/s00221-023-06693-8

[6] Yu, T. et al. EZH2 interacts with HP1BP3 to epigenetically activate WNT7B that promotes temozolomide resistance in glioblastoma. *Oncogene* **42**, 461–470 (2023). 36517590 10.1038/s41388-022-02570-w

[7] Yang, R. et al. E2F7-EZH2 axis regulates PTEN/AKT/mTOR signalling and glioblastoma progression. *Br. J. Cancer* **123**, 1445–1455 (2020). 32814835 10.1038/s41416-020-01032-y PMC7591888

[8] Wong, Y. P. et al. High EZH2 protein expression is a poor prognostic predictor in IDH1 R132H-negative gliomas. *Diagnostics (Basel)* **12**, 2383 (2022). 36292072 10.3390/diagnostics12102383 PMC9600772

[9] Del Moral-Morales, A. et al. EZH2 mediates proliferation, migration, and invasion promoted by estradiol in human glioblastoma cells. *Front. Endocrinol.* **13**, 703733 (2022). 10.3389/fendo.2022.703733 PMC8859835 35197928

[10] Ratnam, N. M. et al. Reversing epigenetic gene silencing to overcome immune evasion in CNS malignancies. *Front. Oncol.* **11**, 719091 (2021). 34336705 10.3389/fonc.2021.719091 PMC8320893

[11] Scuderi, S. A. GSK343, an inhibitor of enhancer of zeste homolog 2, reduces glioblastoma progression through inflammatory process modulation: focus on canonical and non-canonical NF-κB/IκBα pathways. *Int. J. Mol. Sci.* **23**, 13915 (2022). 36430394 10.3390/ijms232213915 PMC9694970

[12] Sun, W. et al. Targeting EZH2 depletes LMP1-induced activated regulatory T cells enhancing antitumor immunity in nasopharyngeal carcinoma. *J. Cancer Res. Ther.* **16**, 309–319 (2020). 32474518 10.4103/jcrt.JCRT_986_19

[13] Qi, L. et al. Evaluation of an EZH2 inhibitor in patient-derived orthotopic xenograft models of pediatric brain tumors alone and in combination with chemo- and radiation therapies. *Lab. Investig.* **102**, 185–193 (2022). 34802040 10.1038/s41374-021-00700-8 PMC10228180

[14] Duan, R., Du, W. & Guo, W. EZH2: a novel target for cancer treatment. *J. Hematol. Oncol.* **13**, 1–12 (2020). 32723346 10.1186/s13045-020-00937-8 PMC7385862

[15] Zeng, D. et al. Blocking EZH2 methylation transferase activity by GSK126 decreases stem cell-like myeloma cells. *Oncotarget* **8**, 3396–3411 (2016). 10.18632/oncotarget.13773 PMC5356890 27926488

[16] Zhai, X. EZH2 regulates the malignancy of human glioblastoma cells via modulation of twist mRNA stability. *Eur. J. Pharmacol.* **904**, 174177 (2021). 34015321 10.1016/j.ejphar.2021.174177

[17] Li, C. et al. Finding an easy way to harmonize: a review of advances in clinical research and combination strategies of EZH2 inhibitors. *Clin. Epigenet.* **13**, 1–12 (2021). 10.1186/s13148-021-01045-1 PMC7992945 33761979

[18] Jardim, D. L. et al. Cyclin pathway genomic alterations across 190,247 solid tumors: leveraging large-scale data to inform therapeutic directions. *Oncologist* **26**, e78–e89 (2021). 32885893 10.1634/theoncologist.2020-0509 PMC7794175

[19] Vij, M. et al. P16 immunohistochemistry is a sensitive and specific surrogate marker for CDKN2A homozygous deletion in gliomas. *Acta Neuropathol. Commun.* **11**, 73 (2023). 37138345 10.1186/s40478-023-01573-2 PMC10155323

[20] Funakoshi, Y. et al. Clinical significance of CDKN2A homozygous deletion in combination with methylated MGMT status for IDH-wildtype glioblastoma. *Cancer Med.* **10**, 3177–3187 (2021). 33838014 10.1002/cam4.3860 PMC8124111

[21] Riess, C. et al. Implementation of a combined CDK inhibition and arginine-deprivation approach to target arginine-auxotrophic glioblastoma multiforme cells. *Cell Death Dis.* **13**, 555 (2022). 35717443 10.1038/s41419-022-05006-1 PMC9206658

[22] Riess, C. et al. Cyclin-dependent kinase inhibitors exert distinct effects on patient-derived 2D and 3D glioblastoma cell culture models. *Cell Death Discov.* **7**, 54 (2021). 33723248 10.1038/s41420-021-00423-1 PMC7961149

[23] Corona, S. P. & Generali, D. Abemaciclib: a CDK4/6 inhibitor for the treatment of HR+/HER2– advanced breast cancer. *Drug Des. Devel. Ther.* **12**, 321–330 (2018). 29497278 10.2147/DDDT.S137783 PMC5818877

[24] Goetz, M. P. et al. MONARCH 3: abemaciclib as initial therapy for advanced breast cancer. *J. Clin. Oncol.* **35**, 3638–3646 (2017). 28968163 10.1200/JCO.2017.75.6155

[25] Wu, T. et al. Effect of abemaciclib (LY2835219) on enhancement of chemotherapeutic agents in ABCB1 and ABCG2 overexpressing cells in vitro and in vivo. *Biochem. Pharmacol.* **124**, 29–42 (2017). 27816545 10.1016/j.bcp.2016.10.015

[26] Bronner, S. M. et al. Design of a brain-penetrant CDK4/6 inhibitor for glioblastoma. *Bioorg. Med. Chem. Lett.* **29**, 2294–2301 (2019). 31307887 10.1016/j.bmcl.2019.06.021

[27] Kellie Turner, P. et al. Abemaciclib does not have a clinically meaningful effect on pharmacokinetics of CYP1A2, CYP2C9, CYP2D6, and CYP3A4 substrates in patients with cancer. *Drug Metab. Dispos.* **48**, 796–803 (2020). 32581049 10.1124/dmd.119.090092

[28] Kaniskan, H. Ü., Martini, M. L. & Jin, J. Inhibitors of protein methyltransferases and demethylases. *Chem. Rev.* **118**, 989–1068 (2018). 28338320 10.1021/acs.chemrev.6b00801 PMC5610952

[29] Liu, W. J. et al. p62 links the autophagy pathway and the ubiqutin-proteasome system upon ubiquitinated protein degradation. *Cell. Mol. Biol. Lett.* **21**, 1–14 (2016). 28536631 10.1186/s11658-016-0031-z PMC5415757

[30] Smith, J., Field, M. & Sugaya, K. Suppression of NANOG expression reduces drug resistance of cancer stem cells in glioblastoma. *Genes* **14**, 1276 (2023). 37372456 10.3390/genes14061276 PMC10297980

[31] Nakod, P. S., Kondapaneni, R. V., Edney, B., Kim, Y. & Rao, S. S. The impact of temozolomide and lonafarnib on the stemness marker expression of glioblastoma cells in multicellular spheroids. *Biotechnol. Prog.* **38**, e3284 (2022). 35768943 10.1002/btpr.3284

[32] Ishii, H. et al. Isolation and characterization of cancer stem cells derived from human glioblastoma. *Am. J. Cancer Res.* **11**, 441 (2021). 33575080 PMC7868757

[33] Figarella-Branger, D., Colin, C., Baeza-Kallee, N. & Tchoghandjian, A. A2B5 expression in central nervous system and gliomas. *Int. J. Mol. Sci.* **23**, 4670 (2022). *2022*, *Vol. 23, Page 4670*. 35563061 10.3390/ijms23094670 PMC9103745

[34] Sun, T. et al. Aggressive invasion is observed in CD133-/A2B5+ glioma-initiating cells. *Oncol. Lett.* **10**, 3399–3406 (2015). 26788141 10.3892/ol.2015.3823 PMC4665828

[35] Noronha, C. et al. Cadherin expression and EMT: a focus on gliomas. *Biomedicines* **9**, 1328 (2021). 34680444 10.3390/biomedicines9101328 PMC8533397

[36] Pinto, J. P. et al. StemChecker: a web-based tool to discover and explore stemness signatures in gene sets. *Nucleic Acids Res.* **43**, W72 (2015). 26007653 10.1093/nar/gkv529 PMC4489266

[37] Zhang, L. et al. EZH2-, CHD4-, and IDH-linked epigenetic perturbation and its association with survival in glioma patients. *J. Mol. Cell Biol.* **9**, 477–488 (2017). 29272522 10.1093/jmcb/mjx056 PMC5907834

[38] Stazi, G. et al. Dissecting the role of novel EZH2 inhibitors in primary glioblastoma cell cultures: effects on proliferation, epithelial-mesenchymal transition, migration, and on the pro-inflammatory phenotype. *Clin. Epigenet.* **11**, 173 (2019). 10.1186/s13148-019-0763-5 PMC6889222 31791385

[39] Straining, R. & Eighmy, W. Tazemetostat: EZH2 inhibitor. *J. Adv. Pract. Oncol.* **13**, 158 (2022). 35369397 10.6004/jadpro.2022.13.2.7 PMC8955562

[40] Liu, H. et al. Therapeutic strategies of glioblastoma (GBM): the current advances in the molecular targets and bioactive small molecule compounds. *Acta Pharm. Sin. B* **12**, 1781–1804 (2022). 35847506 10.1016/j.apsb.2021.12.019 PMC9279645

[41] Ma, G. F. et al. HER2 mRNA status contributes to the discrepancy between gene amplification and protein overexpression in gastric cancer. *Dig. Dis. Sci.* **59**, 328–335 (2014). 24185685 10.1007/s10620-013-2925-1

[42] Cousins, E. M. et al. Competitive kinase enrichment proteomics reveals that abemaciclib inhibits GSK3β and activates WNT signaling. *Mol. Cancer Res.* **16**, 333–344 (2018). 29133594 10.1158/1541-7786.MCR-17-0468 PMC5805620

[43] & Billard-Sandu, C. CDK4/6 inhibitors in P16/HPV16-negative squamous cell carcinoma of the head and neck. *Eur. Arch. Otorhinolaryngol.* **277**, 1273–1280 (2020). 32162057 10.1007/s00405-020-05891-2

[44] Su, D. et al. Identification of predictors of drug sensitivity using patient-derived models of esophageal squamous cell carcinoma. *Nat. Commun.* **10**, 5076 (2019). 31700061 10.1038/s41467-019-12846-7 PMC6838071

[45] Schoenwaelder, N. et al. Preclinical head and neck squamous cell carcinoma models for combined targeted therapy approaches. *Cancers* **14**, 2484 (2022). 35626088 10.3390/cancers14102484 PMC9139292

[46] Dan, S. et al. Correlating phosphatidylinositol 3-kinase inhibitor efficacy with signaling pathway status: in silico and biological evaluations. *Cancer Res.* **70**, 4982–4994 (2010). 20530683 10.1158/0008-5472.CAN-09-4172

[47] Samarzija, I., Tomljanovic, M., Novak Kujundzic, R. & Troselj, K. G. EZH2 inhibition and cisplatin as a combination anticancer therapy: an overview of preclinical studies. *Cancers (Basel)* **14**, 4761 (2022). 36230683 10.3390/cancers14194761 PMC9561994

[48] Daures, M. et al. A new metabolic gene signature in prostate cancer regulated by JMJD3 and EZH2. *Oncotarget* **9**, 23413–23425 (2018). 29805743 10.18632/oncotarget.25182 PMC5955128

[49] Liu, L. et al. Ezh2 promotes mammary tumor initiation through epigenetic regulation of the Wnt and mTORC1 signaling pathways. *Proc. Natl. Acad. Sci. USA* **120**, e2303010120 (2023). 37549258 10.1073/pnas.2303010120 PMC10438390

[50] Shi, J. et al. HOTAIR-EZH2 inhibitor AC1Q3QWB upregulates CWF19L1 and enhances cell cycle inhibition of CDK4/6 inhibitor palbociclib in glioma. *Clin. Transl. Med.* **10**, 182–198 (2020). 32508030 10.1002/ctm2.21 PMC7240863

[51] Kanzawa, T. et al. Role of autophagy in temozolomide-induced cytotoxicity for malignant glioma cells. *Cell Death Differ.* **11**, 448–457 (2004). *2004 114*. 14713959 10.1038/sj.cdd.4401359

[52] Yu, T. et al. The EZH2 inhibitor GSK343 suppresses cancer stem-like phenotypes and reverses mesenchymal transition in glioma cells. *Oncotarget* **8**, 98348–98359 (2017). 29228694 10.18632/oncotarget.21311 PMC5716734

[53] Wang, Z., Zhang, S., Siu, T. L. & Huang, S. Glioblastoma multiforme formation and EMT: role of FoxM1 transcription factor. *Curr. Pharm. Des.* **21**, 1268 (2015). 25506897 10.2174/1381612821666141211115949 PMC4380124

[54] Dong, C. et al. Identification of the proliferative effect of Smad2 and 3 in the TGF β2/Smad signaling pathway using RNA interference in a glioma cell line. *Mol. Med. Rep.* **12**, 1824 (2015). 25891822 10.3892/mmr.2015.3614 PMC4464434

[55] Paredes, F. et al. Metabolic regulation of the proteasome under hypoxia by Poldip2 controls fibrotic signaling in vascular smooth muscle cells. *Free Radic. Biol. Med.* **195**, 283–297 (2023). 36596387 10.1016/j.freeradbiomed.2022.12.098 PMC10268434

[56] Kaundal, B., Karmakar, S. & Roy Choudhury, S. Mitochondria-targeting nano therapy altering IDH2-mediated EZH2/EZH1 interaction as precise epigenetic regulation in glioblastoma. *Biomater. Sci.* **10**, 5301–5317 (2022). 35917200 10.1039/D1BM02006D

[57] D’Angiolella, V. et al. Cyclin F-mediated degradation of ribonucleotide reductase M2 controls genome integrity and DNA repair. *Cell* **149**, 1023–1034 (2012). 22632967 10.1016/j.cell.2012.03.043 PMC3616325

[58] Perrault, E. N. et al. Ribonucleotide reductase regulatory subunit M2 drives glioblastoma TMZ resistance through modulation of dNTP production. *Sci. Adv.* **9**, eade7236 (2023). 37196077 10.1126/sciadv.ade7236 PMC10191446

[59] Corrales-Guerrero, S. et al. Inhibition of RRM2 radiosensitizes glioblastoma and uncovers synthetic lethality in combination with targeting CHK1. *Cancer Lett.* **570**, 216308 (2023). 37482342 10.1016/j.canlet.2023.216308

[60] Liu, Y. et al. DNA topoisomerase II alpha promotes the metastatic characteristics of glioma cells by transcriptionally activating β-catenin. *Bioengineered* **13**, 2207 (2022). 35012441 10.1080/21655979.2021.2023985 PMC8974225

[61] Kong, W. Z., Liu, Y. S., Gao, X. D. & Fujita, M. Comprehensive in silico analysis of glycosylphosphatidylinositol- anchored protein (GPI-AP) related genes expression profiles in human normal and cancer tissues. *Yi Chuan Hered.* **45**, 669–683 (2023). 10.16288/j.yczz.23-043 37609818

[62] Eyme, K. M. et al. Targeting de novo lipid synthesis induces lipotoxicity and impairs DNA damage repair in glioblastoma mouse models. *Sci. Transl. Med.* **15**, eabq6288 (2023). 36652537 10.1126/scitranslmed.abq6288 PMC9942236

[63] Zhang, X. et al. PLK4 initiates crosstalk between cell cycle, cell proliferation and macrophages infiltration in gliomas. *Front. Oncol.* **12**, 1055371 (2022). 36620611 10.3389/fonc.2022.1055371 PMC9815703

[64] Pei, J. P. et al. AXL antibody and AXL-ADC mediate antitumor efficacy via targeting AXL in tumor-intrinsic epithelial-mesenchymal transition and tumor-associated M2-like macrophage. *Acta Pharmacol. Sin.* **44**, 1290–1303 (2023). 36650292 10.1038/s41401-022-01047-6 PMC10203350

[65] Walentynowicz, K. A. et al. Single-cell heterogeneity of EGFR and CDK4 co-amplification is linked to immune infiltration in glioblastoma. *Cell Rep.* **42**, 112235 (2023). 36920905 10.1016/j.celrep.2023.112235 PMC10114292

[66] Chen, X., Hu, Y., Wang, S. & Sun, X. The regulator of calcineurin 1 (RCAN1) inhibits nuclear factor kappaB signaling pathway and suppresses human malignant glioma cells growth. *Oncotarget* **8**, 12003–12012 (2017). 28061453 10.18632/oncotarget.14479 PMC5355321

[67] Liu, B. et al. RND3 promotes Snail 1 protein degradation and inhibits glioblastoma cell migration and invasion. *Oncotarget* **7**, 82411–82423 (2016). 27705942 10.18632/oncotarget.12396 PMC5347701

[68] Salewski, I. et al. CDK4/6 blockade provides an alternative approach for treatment of mismatch-repair deficient tumors. *Oncoimmunology* **11**, 2094583 (2022). 35845723 10.1080/2162402X.2022.2094583 PMC9278458

[69] Strüder, D. et al. Establishment and characterization of patient-derived head and neck cancer models from surgical specimens and endoscopic biopsies. *J. Exp. Clin. Cancer Res.* **40**, 246 (2021). 34362423 10.1186/s13046-021-02047-w PMC8344210

[70] Koczan, D., Fitzner, B., Zettl, U. K. & Hecker, M. Microarray data of transcriptome shifts in blood cell subsets during S1P receptor modulator therapy. *Sci. Data* **5**, 180145 (2018). 30040082 10.1038/sdata.2018.145 PMC6057441

[71] Hong, G., Zhang, W., Li, H., Shen, X. & Guo, Z. Separate enrichment analysis of pathways for up- and downregulated genes. *J. R. Soc. Interface* **11**, 20130950 (2014). 24352673 10.1098/rsif.2013.0950 PMC3899863

[72] Chicco, D. & Agapito, G. Nine quick tips for pathway enrichment analysis. *PLoS Comput. Biol.* **18**, e1010348 (2022). 35951505 10.1371/journal.pcbi.1010348 PMC9371296

[73] Szklarczyk, D. et al. The STRING database in 2021: customizable protein–protein networks, and functional characterization of user-uploaded gene/measurement sets. *Nucleic Acids Res.* **49**, D605 (2021). 33237311 10.1093/nar/gkaa1074 PMC7779004

[74] Raudvere, U. et al. g:Profiler: a web server for functional enrichment analysis and conversions of gene lists (2019 update). *Nucleic Acids Res.* **47**, W191–W198 (2019). 31066453 10.1093/nar/gkz369 PMC6602461

[75] Maletzki, C. et al. Establishing safe high hydrostatic pressure devitalization thresholds for autologous head and neck cancer vaccination and reconstruction. *Cell Death Discov.* **9**, 390 (2023). 37872173 10.1038/s41420-023-01671-z PMC10593744

[76] Linke, C. et al. The addition of arginine deiminase potentiates mithramycin a-induced cell death in patient-derived glioblastoma cells via ATF4 and cytochrome C. *Cancer Cell Int*. **23**, 38 (2023). 36843002 10.1186/s12935-023-02873-2 PMC9969664

[77] Jacob, F. et al. A patient-derived glioblastoma organoid model and biobank recapitulates inter- and intra-tumoral heterogeneity. *Cell* **180**, 188–204.e22 (2020). 31883794 10.1016/j.cell.2019.11.036 PMC7556703

