# Supplementary material for: Combined inhibition of EZH2 and CDK4/6 perturbs endoplasmic reticulum-mitochondrial homeostasis and increases antitumor activity against glioblastoma
Source: NPJ Precis Oncol. 2024 Jul 25;8:156. doi: 10.1038/s41698-024-00653-3 (PMC11272933; doi:10.1038/s41698-024-00653-3)
Supplement: Supplementary file 1 — Supplementary figures with legends beneath each one [file 41698_2024_653_MOESM1_ESM.pdf]

Supplementary files

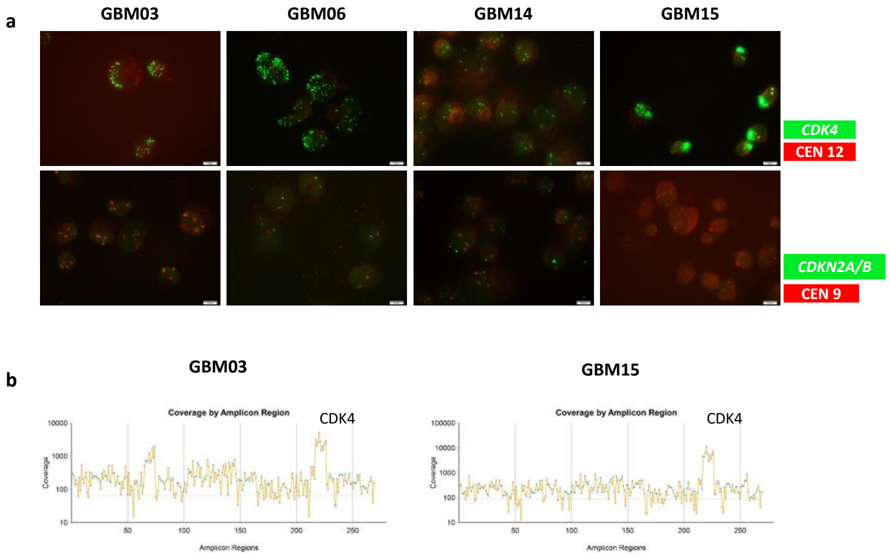

Supplementary Figure 1: Molecular analysis. (A) FISH analysis for *CDK4* and *CDKN2A* of four patient-derived GBM cell lines and (B) Next-generation sequencing results showing *CDK4* amplification in GBM03 and GBM15.

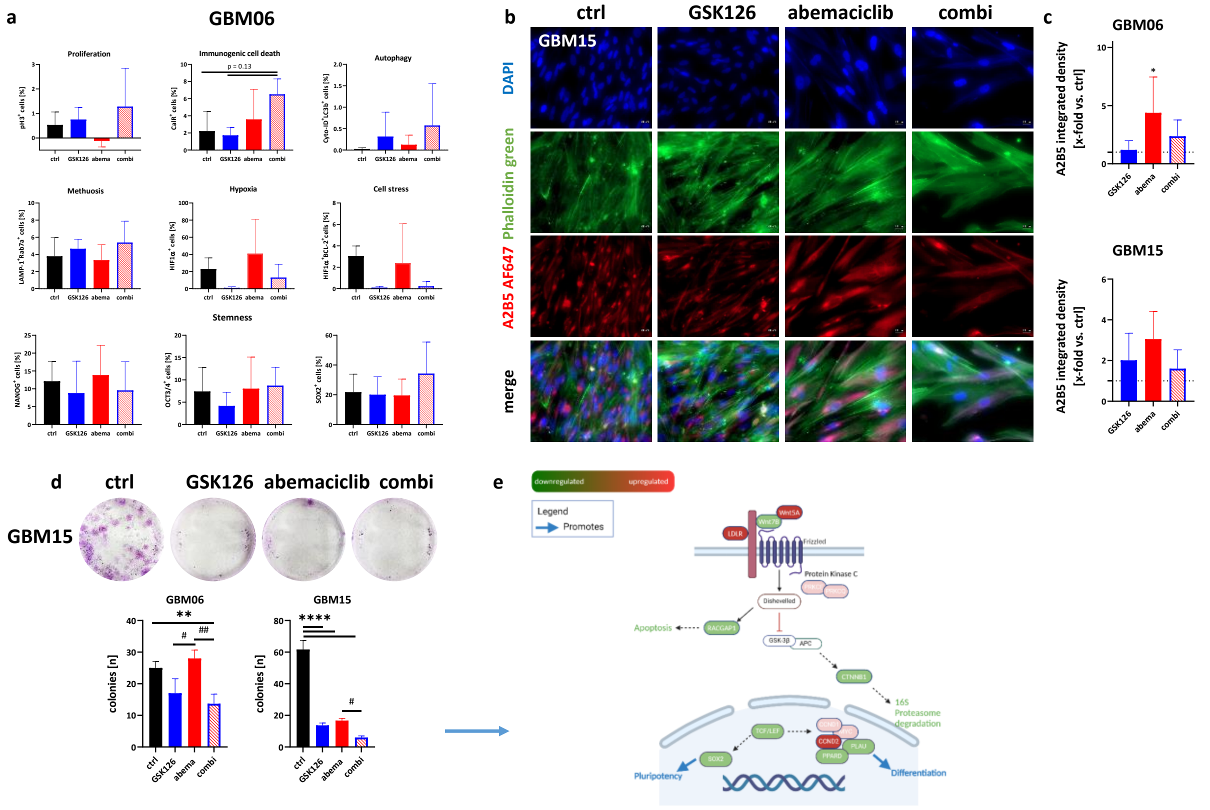

Supplementary Figure 2: Influence on autophagy, proliferation, cell stress, methuosis, and stemness. (A) Quantitative analysis of the investigated markers by spectral flow cytometry in GBM06 cells. Given is the % of positive cells after exposure to GSK126 (10 µM), abemaciclib (1 µM) and the combination of both after 1 x 72h; n = 3-5, mean ± s.d. (B, C) A2B5 immunofluorescence for detection of stemness phenotypes in GBM cells (GBM06, GBM15). (B) Single channel and merged fluorescence is presented (representative images for GBM15 are shown, scale bar A: 20 µm). Analysis was done after 2 x 72h treatment with GSK126 (10 µM), abemaciclib (1 µM), or the combination of both. Nuclei were stained with DAPI (blue), A2B5 was detected by using an Alexa Fluor® 647 anti-A2B5 antibody. (C) The quantification is presented as the x-fold change (integrated density) relative to the control (DMSO), which was set to =1 (dotted line); n = 3, mean ± s.d. One-way ANOVA (Tukey's multiple comparisons test); * p<0.05 (comparison between control and test group). (D) Colony formation assay. The upper images show representative pictures of GBM15 exposed to GSK126 (10 µM), abemaciclib (1 µM), or the combination of both. DMSO-treated cells served as controls. The lower part shows the quantitative data of GBM06 and GBM15. Colony forming ability was reduced after GSK126 mono- and combination

treatment, while in GBM06, it was highest after abemaciclib, indicating enrichment of stem-like cells. (E) Pathway regulation identified based on microarray data obtained from GBM15 spheroids. Essential genes involved in Wnt signaling were significantly downregulated, confirming the reversal of stem-like characteristics upon dual EZH2/CDK4/6 blockade. Green - downregulated genes; red – upregulated genes. Created with Biorender.com.

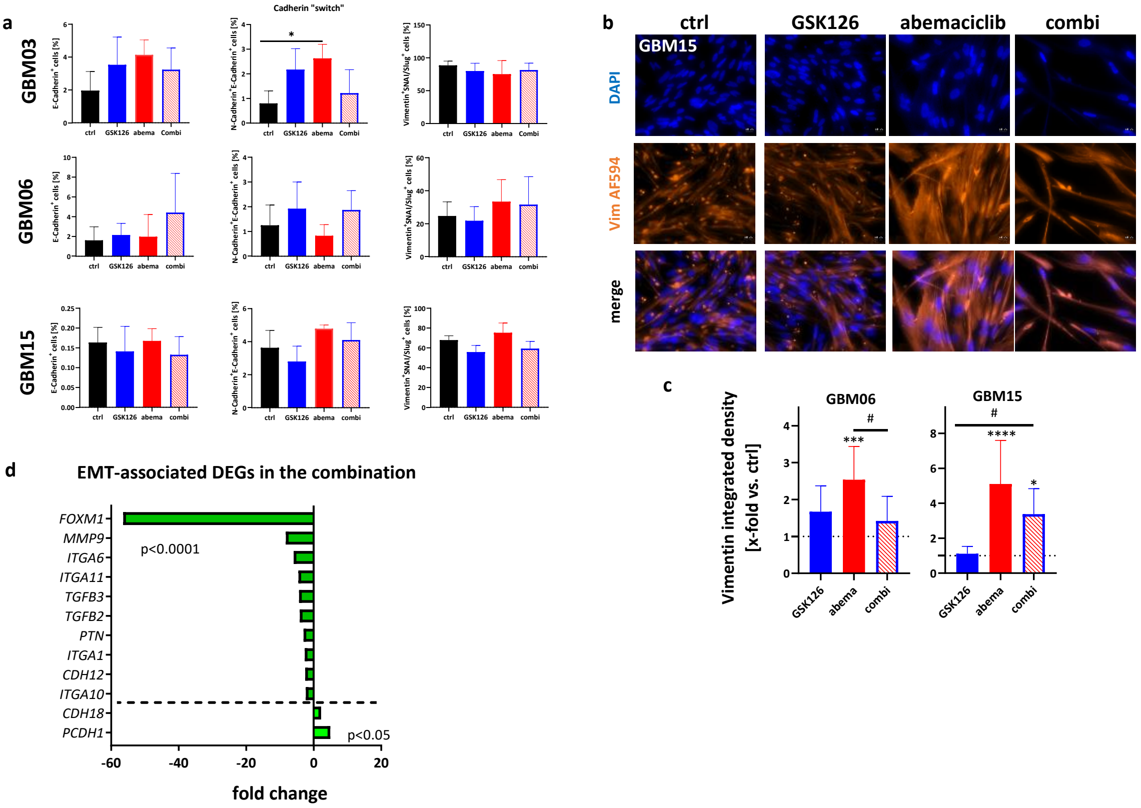

Supplementary Figure 3: Impact on epithelial-mesenchymal transition marker. (A) Quantitative analysis of the investigated markers by spectral flow cytometry. Given is the % of positive cells after exposure to GSK126 (10 µM), abemaciclib (1 µM) and the combination of both after 1 x 72h; n = 3-5, mean ± s.d. One-way ANOVA (Tukey's multiple comparisons test); *p<0.05 (comparison between control and testing group). (B, C) Vimentin immunofluorescence for EMT analysis in GBM cells (GBM06, GBM15). (B) Single channel and merged fluorescence is presented (representative images for GBM15 are shown, scale bar A: 20 µm). Analysis was done after 2 x 72h treatment with GSK126 (10 µM), abemaciclib (1 µM), or the combination of both. Nuclei were stained with DAPI (blue), vimentin was detected by using an Alexa Fluor® 546 anti-vimentin antibody. (C) The quantification is presented as the x-fold change (integrated density) relative to the control (DMSO), which was set to =1 (dotted line); n = 3, mean ± s.d. One-way ANOVA (Tukey's multiple comparisons test); * p<0.05; *** p<0.001; **** p<0.0001 (comparison between control and test group); # p<0.05 (comparison between testing groups). (D) Horizontal waterfall chart showing epithelial-mesenchymal transition (EMT)-associated deregulated genes in GBM15 spheroids treated with the combination (GSK126, 10 µM, abemaciclib, 1 µM), 2 x 72h). Microarray analysis. The dotted line separates the downregulated genes from the

upregulated genes. Among the upregulated genes, the tumor suppressor and EMT-suppressor *protocadherin 1 (PCDH1*) was significantly upregulated, while genes associated with an EMT phenotype were significantly downregulated (e.g. *FOXM1* and *MMP9*).

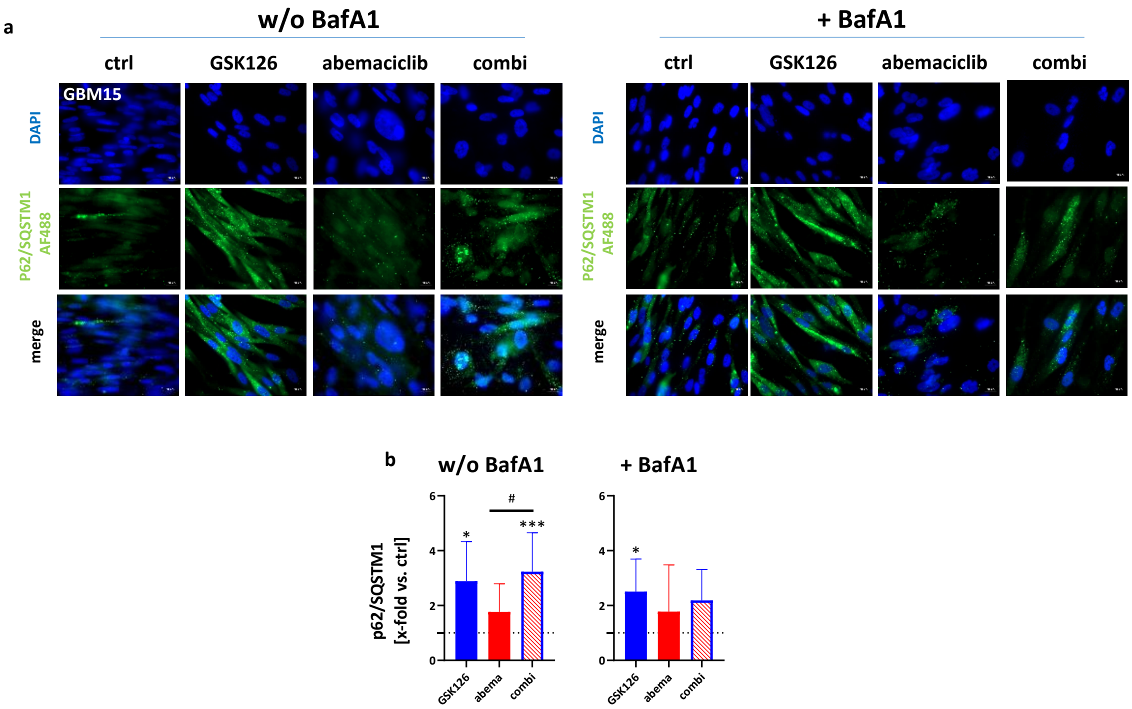

Supplementary Figure 4: Relevance of autophagy. (A) SQSTM1 immunofluorescence for autophagy analysis in GBM15 cells. Single channel and merged fluorescence is presented (representative images for GBM15 are shown, scale bar A: 20 µm). Analysis was done after 2 x 72h treatment with GSK126 (10 µM), abemaciclib (1 µM), or the combination of both. Nuclei were stained with DAPI (blue), SQSTM1 was detected by using an Alexa Fluor® 488 anti- SQSTM1 antibody. (B) The quantification is presented as the x-fold change (integrated density) relative to the control (DMSO), which was set to =1 (dotted line); n = 3, mean ± s.d. One-way ANOVA (Tukey's multiple comparisons test); * $p<0.05$; *** $p<0.001$ (comparison between control and test group); # $p<0.05$ (comparison between testing groups).

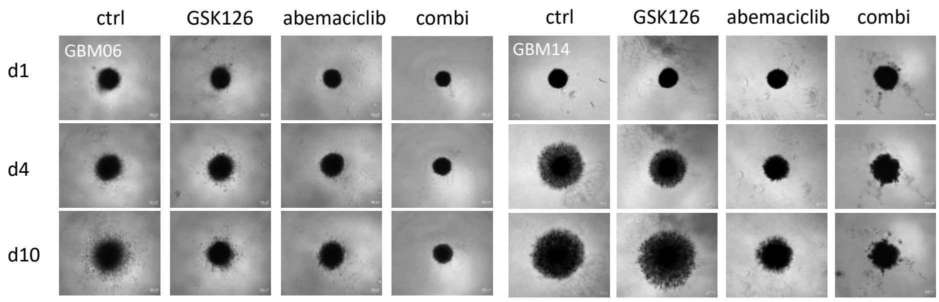

Supplementary Figure 5: Invasion capability of GBM06 and GBM14 cell lines. Representative images of the invasion under treatment with GSK126 (10 µM), abemaciclib (1 µM), the combination of both or with vehicle control after the spheroids were embedded in matrigel. Images were taken on days 1, 4 and 10. The spheroids were treated on day 0 and day 3 (2 x 72h in total).

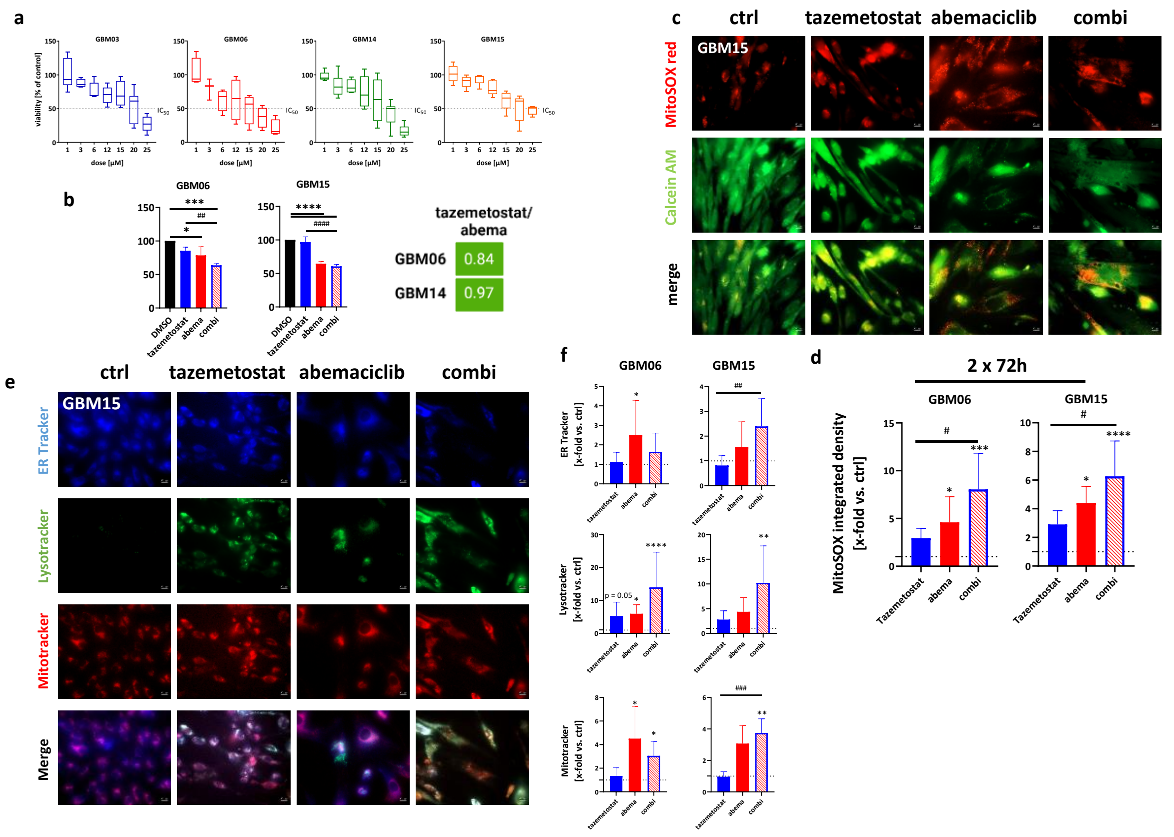

Supplementary Figure 6: Combination approach on GBM06 and GBM15 cells with the FDA-approved EZH2i tazemetostat and abemaciclib. (A) Concentration-response relationships to tazemetostat for two (2x 72h) treatment cycles to determine the IC_{20} and IC_{50} concentrations. (B) Combination approach with tazemetostat (10 µM) and abemaciclib (1 µM) after 2 treatment cycles with 72 hours each; n = 4, mean ± s.d. One-way ANOVA (Tukey's multiple comparisons test); *p<0.05; ***p<0.001; ****p<0.0001 (comparison between control and test group); ## p<0.001; #### p<0.0001 (comparison between testing groups), right site: Bliss independence calculation as described before. Created with Biorender.com. (C, D) MitoSOX™ red was used as an indicator of ROS production in GBM cells. Cells were counterstained with Calcein AM [green] for the spatial distribution. (C) Single channel and merged fluorescence is presented (representative images for GBM15 are shown, scale bar A: 20 µm). Analysis was done after 1 x and 2 x 72h treatment with tazemetostat (10 µM), abemaciclib (1 µM), or the combination of both. (D) Quantification determined as the x-fold change (integrated density) in relation to the control, which was set to be =1 (dotted line); n = 3, mean ± s.d. Kruskal-Wallis test (Dunn's multiple comparisons test); * p<0.05; *** p<0.001; **** p<0.0001 (comparison between

control and test group); # $p<0.05$ (comparison between testing groups); There was a significant time-dependent increase in ROS production when GBM cells were exposed to the tazemetostat/abemaciclib combination. (E, F) Impact on ER stress, lysosomes, and mitochondria. (E) ER stress (ER tracker), acidic compartments (LysoTracker), and mitochondrial function (Mitotracker) were examined in 2D-cultured GBM cells with or without treatment (tazemetostat 10 µM, abemaciclib 1 µM, combination, 2 x 72h) by immunofluorescence staining as described in materials and methods (ER- [blue], Lyso- [green], and Mitotracker [red]). (E) Single channel and merged fluorescence is presented (representative images are shown, scale bar A: 20 µm, n=3). (F) Quantification determined as the x-fold change (integrated density) in relation to the control, which was set to be =1 (dotted line); Kruskal-Wallis test (Dunn's multiple comparisons test); * $p<0.05$; ** $p<0.01$; **** $p<0.0001$ (comparison between control and test group); ## $p<0.01$; ### $p<0.01$ (comparison between testing groups); Complex cellular stress responses were seen in both cell lines.

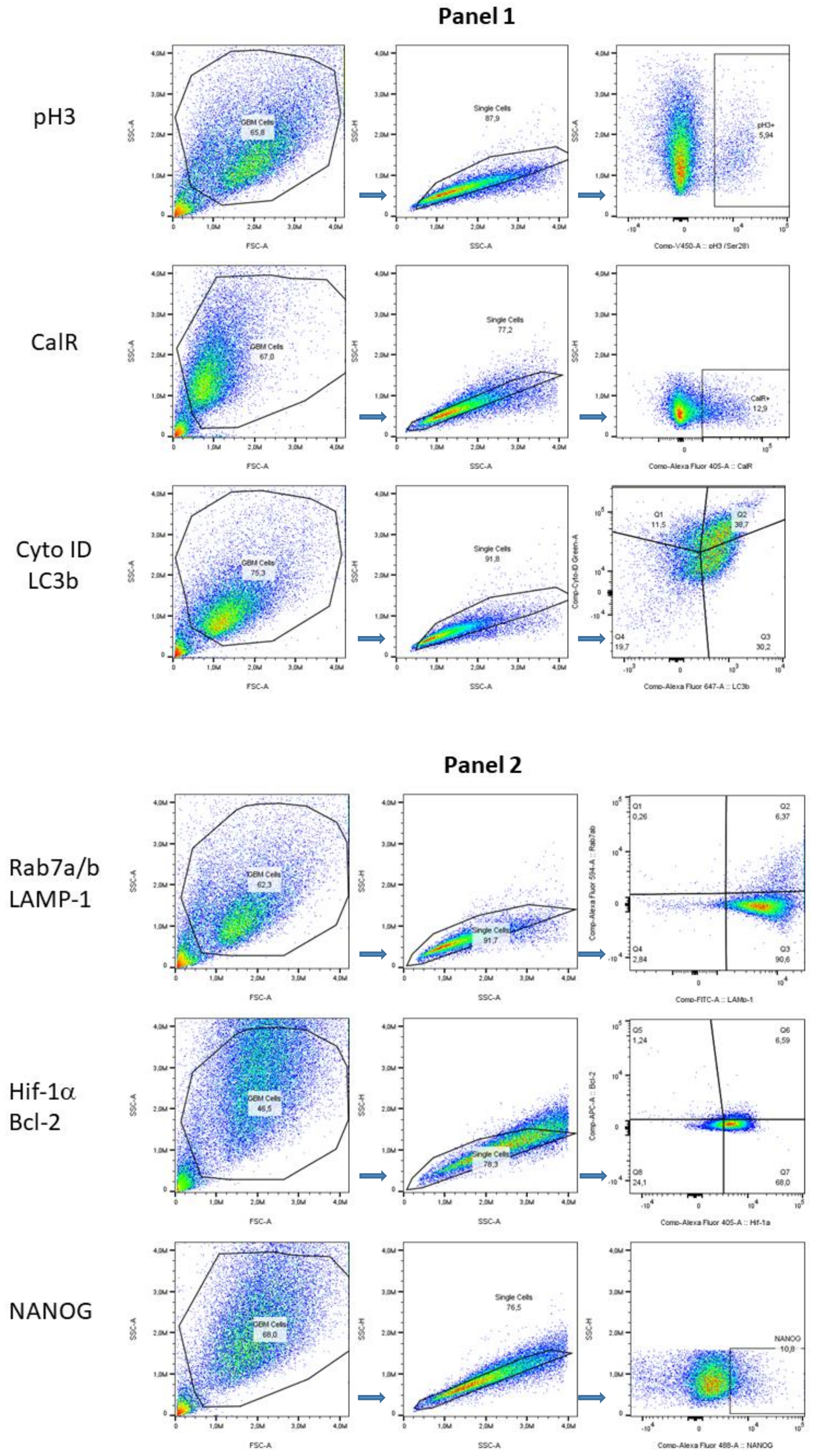

Panel 1
pH3
CalR
Cyto ID
LC3b
GBM Cells
65,8
67,0
75,3
Single Cells
87,9
77,2
91,8
pH3+
5,94
CalR+
12,9
Q1
11,5
Q2
38,7
Q3
30,2
Q4
19,7
SSC-A
SSC-H
FSC-A
Comp-V450-A :: pH3 (Ser28)
Comp-Alexa Fluor 405-A :: CalR
Comp-Alexa Fluor 647-A :: LC3b
Comp-Cyto-ID Green-A
Panel 2
Rab7a/b
LAMP-1
Hif-1α
Bcl-2
NANOG
GBM Cells
62,3
46,5
68,0
Single Cells
91,7
78,3
76,5
Q1
0,26
Q2
6,37
Q3
90,6
Q4
2,84
Q5
1,24
Q6
6,59
Q7
68,0
Q8
24,1
NANOG
10,8
Comp-Alexa Fluor 594-A :: Rab7ab
Comp-FITC-A :: LAMp-1
Comp-APC-A :: Bcl-2
Comp-Alexa Fluor 405-A :: Hif-1a
Comp-Alexa Fluor 488-A :: NANOG

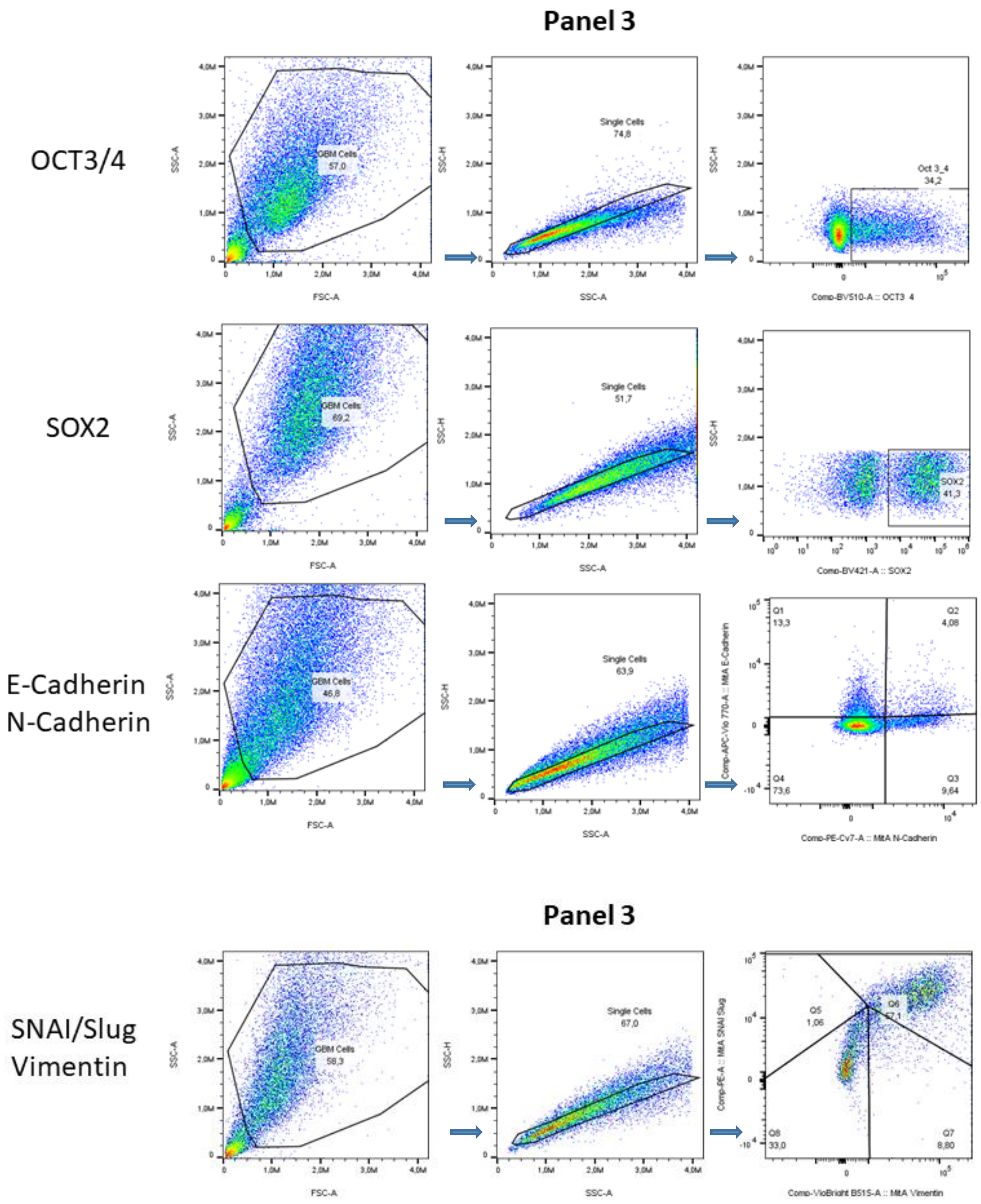

Supplementary Figure 7: Gating strategy for spectral flow cytometry. The detailed gating strategy is shown for the three in-house flow cytometry panels. The cells were identified by FSC/SSC gating (left), followed by exclusion of duplicates (middle) and identification of specific antibody-stained cells (right). Measurements were done using a spectral flow cytometer (3L-Cytek™ Aurora). The data were analyzed using SpectroFlo™ Version 3.2.1. and FlowJo™ Version 10.6.1, respectively.
